# AMPK and Pulmonary Hypertension: Crossroads Between Vasoconstriction and Vascular Remodeling

**DOI:** 10.3389/fcell.2021.691585

**Published:** 2021-06-08

**Authors:** Qiang Zhao, Ping Song, Ming-Hui Zou

**Affiliations:** Center for Molecular and Translational Medicine, Georgia State University, Atlanta, GA, United States

**Keywords:** AMPK, pulmonary hypertension, pulmonary vascular remodeling, hypoxic pulmonary vasoconstriction, metformin

## Abstract

Pulmonary hypertension (PH) is a debilitating and life-threatening disease characterized by increased blood pressure within the pulmonary arteries. Adenosine monophosphate-activated protein kinase (AMPK) is a heterotrimeric serine-threonine kinase that contributes to the regulation of metabolic and redox signaling pathways. It has key roles in the regulation of cell survival and proliferation. The role of AMPK in PH is controversial because both inhibition and activation of AMPK are preventive against PH development. Some clinical studies found that metformin, the first-line antidiabetic drug and the canonical AMPK activator, has therapeutic efficacy during treatment of early-stage PH. Other study findings suggest the use of metformin is preferentially beneficial for treatment of PH associated with heart failure with preserved ejection fraction (PH-HFpEF). In this review, we discuss the “AMPK paradox” and highlight the differential effects of AMPK on pulmonary vasoconstriction and pulmonary vascular remodeling. We also review the effects of AMPK activators and inhibitors on rescue of preexisting PH in animals and include a discussion of gender differences in the response to metformin in PH.

## Introduction

Pulmonary hypertension (PH) is a disease characterized by high blood pressure that affects the vessels in lungs. These changes result in right ventricular failure and ultimately, premature death (Maron and Leopold, 2015). Adenosine monophosphate-activated protein kinase (AMPK) is a central regulator of energy homeostasis. It is activated under a variety of conditions, including hypoxia, nutrient starvation, and toxin exposure (Towler and Hardie, 2007; Kim et al., 2016; Herzig and Shaw, 2018). AMPK exerts most of its biological effects *via* catalytic α-subunits (α1 and α2) that are ubiquitously expressed in pulmonary vessels (Mihaylova and Shaw, 2011; Hardie, 2013; Kim et al., 2016). AMPK α1 is the predominant subunit in small pulmonary artery-derived pulmonary microvascular endothelial cells (ECs) and vascular smooth muscle cells (VSMCs). AMPK α2 is the predominant subunit in conduit pulmonary artery-derived ECs and VSMCs (Evans et al., 2005; Creighton et al., 2011). The AMPK α1 and AMPK α2 subunits have different effects on survival of pulmonary VSMCs and hypoxic pulmonary vasoconstriction. For example, activation of AMPK α1 stimulates autophagy in pulmonary artery VSMCs, but AMPK α2 activation prevents apoptosis (Ibe et al., 2013). Under conditions of mild hypoxia, AMPK α1 is activated by liver kinase B1 (LKB1) and is required for hypoxic pulmonary vasoconstriction; the AMPK α2 subunit is required under conditions of severe hypoxia (Moral-Sanz et al., 2018). Because of these characteristics, studies found that use of AMPK-targeting agonists and antagonists results in contradictory effects on PH development. Some studies found that AMPK activators [i.e., metformin (Agard et al., 2009; Dean et al., 2016; Lai et al., 2016; Omura et al., 2016; Zhai et al., 2018; Zhang et al., 2018; Wang et al., 2020), 5-aminoimidazole-4-carboxamide (AICAR) (Huang et al., 2014; Chen et al., 2016; Dean et al., 2016), rosiglitazone/pioglitazone (Hansmann et al., 2007; Satoh et al., 2009; Kim et al., 2010; Legchenko et al., 2018), and apelin (Chandra et al., 2011; Kim, 2014)] are protective against experimental PH. Other studies found that AMPK activation induces hypoxic pulmonary vasoconstriction (Evans, 2006; Robertson et al., 2008; Evans et al., 2009; Moral-Sanz et al., 2018) and that inhibition of AMPK by compound C prevents PH (Ibe et al., 2013). Results from human clinical studies are not currently conclusive on the precise role of AMPK in PH because studies on PH treatment using metformin are currently phase two clinical trials (NCT01884051 and NCT03629340). The AMPK paradox remains relevant.

## AMPK: Structure and Regulation

### AMPK Structure

**FIGURE 1:**
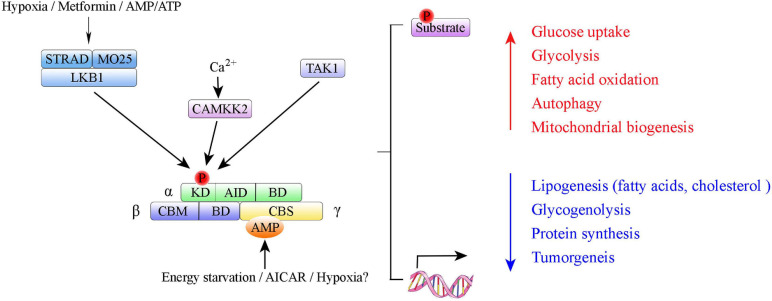
Summary of AMP-activated protein kinase (AMPK) structure and activation. Domain structure of AMPK trimer: α-, β-, and γ-subunits with respective domains. AMPK α subunits: KD, kinase domain containing Thr-172 phosphorylation site; AID, autoinhibitory domain; BD, binding domain. AMPK β subunits: CBM, carbohydrate binding module; BD, binding domain. AMPK γ subunits: CBS, cystathione-β-synthase domain. The upstream kinases LKB1, CAMKK2, and TAK1 are shown above the AMPK complex. LKB1 in complex with STRAD and MO25 activates AMPK; CAMKK2 activated by intracellular calcium.

Adenosine monophosphate-activated protein kinase is a highly conserved serine/threonine protein kinase complex consisting of a catalytic α-subunit, a scaffolding β-subunit, and a regulatory γ-subunit (Figure 1). In eukaryotes, each subunit has multiple distinct isoforms encoded by different genes. The α-subunit has two isoforms, α1 and α2, encoded by genes *Prkaa1* and *Prkaa2*, respectively (Stapleton et al., 1996). It contains a canonical N-terminal Ser/Thr kinase domain (KD), an auto-inhibitory domain (AID), and an adenine nucleotide sensor segment termed an α-linker (Herzig and Shaw, 2018; Yan et al., 2018). AMPK activation requires phosphorylation of critical residues (Thr174 in the AMPK α1 subunit and Thr172 in the AMPK α2 subunit) within the activation loop of the KD in the AMPKα catalytic subunit that is phosphorylated by upstream kinases LKB1 (Hudson et al., 2003), Ca^2+/^calmodulin-dependent protein kinase β (CaMKKβ) (Woods et al., 2005), or TGF-beta-activated kinase-1 (TAK-1) (Momcilovic et al., 2006). AMPK auto-inhibition requires an AID, which interacts with the KD and causes AMPK to be maintained as an inactive conformation (Chen et al., 2013; Kim et al., 2016). The β-subunit also has two isoforms, β1 and β2, encoded by *Prkab1* and *Prkab2*, respectively (Hudson et al., 2003). The γ-subunit has three isoforms, γ1, γ2, and γ3, encoded by *Prkag1*, *Prkag2*, and *Prkag3*, respectively (Cheung et al., 2000). The γ-subunits contain four tandem cystathionine-β-synthase domains, which enable AMP, ADP, or ATP binding (Xiao et al., 2007). Binding of AMP, and to a lesser extent ADP, to the γ-subunit is an important regulatory feature of the conformational switch that activates the AMPK complex (Hardie et al., 2011; Gowans et al., 2013; Ross et al., 2016a). Each AMPK complex consists of one α-subunit, one β-subunit, and one γ-subunit, and all 12 heterotrimeric combinations are possible (Ross et al., 2016b). Different subunits have distinct organ preferences and expression patterns. For example, the AMPK α1 subunit is mainly expressed in adipose tissue (Ruderman et al., 2003; Rutter et al., 2003; Kelly et al., 2004). The AMPK α2 subunit is predominantly expressed in skeletal muscle and cardiac myocytes (Sakamoto et al., 2005, 2006; Thomson et al., 2007). Isoform-specific roles of AMPK α1/AMPK α2 contribute to the pathogenesis of different diseases (e.g., cardiovascular disease (Ahmad et al., 2005; Sakamoto et al., 2006; Zarrinpashneh et al., 2006; Arad et al., 2007), osteoclastogenesis (Wang et al., 2016), and Alzheimer’s disease (Zhao et al., 2020)).

### AMPK Signaling Transduction

Adenosine monophosphate-activated protein kinase can be phosphorylated directly by small molecules that mimic cellular decreased ATP-to-ADP or ATP-to-AMP ratios or three upstream AMPK kinases (i.e., LKB1, CaMKKβ and TAK1) (Figure 1). Upon changes in ATP/ADP and ATP/AMP ratios that occur during nutrient starvation, AMP binds to the AMPK γ subunit to cause allosteric activation *via* modulation of the phosphorylation state of Thr172 (Xiao et al., 2011; Oakhill et al., 2012; Gowans et al., 2013). LKB1, in a complex with the pseudokinase STRAD and the scaffolding protein MO25, directly phosphorylates AMPK at Thr172 (Lizcano et al., 2004). Study results indicate that LKB1 is the principal route *via* which AMPK is activated in many organs (e.g., skeletal muscle, adipose tissue, and liver) (Shackelford and Shaw, 2009). Whereas, CaMKKβ activates AMPK in response to Ca^2+^ signaling pathways (Hawley et al., 2005; Hong et al., 2005; Hurley et al., 2005). In 2006, TAK1 (i.e., MAPKK kinase-7, MAP3K7) was identified as the third kinase capable of direct AMPK activation (Momcilovic et al., 2006).

Adenosine monophosphate-activated protein kinase can be activated by hypoxia in various tissue and cell types (Mungai et al., 2011; Sallé-Lefort et al., 2016), but long-term hypoxia exposure inhibits AMPK activation (de Theije et al., 2018). Many studies found that activation of AMPK under hypoxia primarily implies LKB1 activity, because AMPK activation is abrogated in LKB1-deleted cells and knockout of CaMKK2, or another upstream kinase, has no effect on AMPK activation in VSMCs under hypoxic conditions (Moral-Sanz et al., 2018). Some studies found that LKB1 seems to only activate the AMPK α2 subunit, because the AMPK α1 subunit remains phosphorylated in LKB1-deficient heart muscle cells (Sakamoto et al., 2006). This result might be explained by differences in abundances and preferences of the AMPK upstream kinase in different cells and organs. Increased production of reactive oxygen species (ROS) in hypoxic conditions contributes to activation of AMPK (Choi et al., 2001; Emerling et al., 2009; Zmijewski et al., 2010; Hinchy et al., 2018). Hypoxia-inducible factor-prolyl-4-hydroxylases (HIF-P4Hs) have a role in the activation of AMPK (Yan et al., 2012; Dengler and Gäbel, 2019).

Once activated, AMPK phosphorylates key proteins in multiple pathways (Marsin et al., 2000; Inoki et al., 2003; Gwinn et al., 2008) or directly regulates key enzymes involved in these pathways. These processes occur over time *via* targeting of transcriptional regulators (Koo et al., 2005; Lamia et al., 2009; Bungard et al., 2010; Figure 1). The most important aspect of AMPK biology is its role in maintaining the balance between catabolism and anabolism in response to metabolic stress (Towler and Hardie, 2007; Hardie, 2008; Fogarty and Hardie, 2010). Studies have revealed the roles of AMPK in lipid homeostasis [e.g., acetyl-CoA carboxylase (Munday et al., 1988) and HMG-CoA reductase (Carling et al., 1987)], glucose metabolism [e.g., thioredoxin-interacting protein (TXNIP) (Wu et al., 2013) and 6-phosphofructo-2-kinase (Bando et al., 2005)], insulin signaling (Galic et al., 2011; Li Y. et al., 2011; Fullerton et al., 2013; Wu et al., 2015; Emilio et al., 2016; Myers et al., 2017), and food intake and body weight (Kahn et al., 2005; Kola et al., 2005; Kola, 2008). Given those functional attributes in metabolism, AMPK is a major therapeutic target for treatment of metabolic diseases (e.g., type 2 diabetes) and obesity (Viollet et al., 2009; Rojas et al., 2011; Hardie, 2013; Day et al., 2017). A growing body of evidence also points to specific regulation of AMPK and mitochondrial homeostasis, including *via* stimulation of mitochondrial biogenesis (Bergeron et al., 2001; Zong et al., 2002; Garcia-Roves et al., 2008), regulation of mitochondrial dynamics (Ducommun et al., 2015; Toyama et al., 2016), and mitophagy (Wang et al., 2001; Egan et al., 2011).

### AMPK and Cardiovascular Disease

Adenosine monophosphate-activated protein kinase has pivotal roles in cardiovascular physiology and in cardiovascular disease states. AMPK α1 is the predominant subunit in VSMCs, ECs, monocytes/macrophages, and adipocytes. AMPK α2 is the predominant subunit in cardiomyocytes (Shirwany and Zou, 2010; Wu and Zou, 2020). The functions of AMPK in cardiovascular disease include contributions to atherosclerosis and to heart failure and hypertension, which have been extensively reviewed elsewhere (Shirwany and Zou, 2010; Wu and Zou, 2020).

## Pulmonary Hypertension

### Categories

Pulmonary hypertension is a general term used to describe increased blood pressure (mean pulmonary arterial pressure, mPAP, exceeds 25 mmHg at rest) in the lungs (Galiè et al., 2009). At the 5th and 6th World Symposium on PH, it was classified into five groups: pulmonary artery hypertension (PAH, Group 1), PH associated with left heart disease (Group 2), PH associated with lung disease and/or hypoxia (Group 3), PH associated with chronic thromboembolic disease (Group 4), and PH with unclear or multifactorial mechanisms, or both (Group 5) (Galiè and Simonneau, 2013; Simonneau et al., 2019). Each group represents a very broad spectrum of disease etiology, pathobiology, hemodynamic characteristics, and therapeutic approaches (Table 1). The detailed features and treatments of pulmonary hypertensive vascular disease in humans have been reviewed elsewhere (Maron and Galiè, 2016; Thenappan et al., 2018). AMPK deficiency has been identified in metabolic syndrome-associated PH (PH-HFpE) (Lai et al., 2016). However, in PAH, AMPK activity and expression can be either inhibited or promoted depending on cell type and branch pulmonary artery diameter (Ibe et al., 2013; Omura et al., 2016; Zhang et al., 2018), which is discussed in section “Clinical Trials of Pulmonary Hypertension Treatment Using Metformin.”

**TABLE 1:** Animal models of pulmonary hypertension.

Cause	Histological features	Animal models
**Group 1: Pulmonary arterial hypertension (PAH)**		
Idiopathic PAH	Pulmonary artery intimal proliferation	Su-Hx rat/mouse
Heritable PAH	Pulmonary artery medical hypertrophy	MCT rat
Drugs/Toxin/Others	Plexiform lesions	Su-Hx-Normoxia
**Group 2: Pulmonary hypertension with left heart disease**		
Left-sided heart disease	Pulmonary medical hypertrophy	SU5416/Obese ZSF1 rat
	Pulmonary vein arterialization	
	Pulmonary interstitial edema	
**Group 3: Pulmonary hypertension associated with lung disease**		
**and/or hypoxemia**		
High altitudes	Hypoxic pulmonary vasoconstriction	Su-Hx rat/mouse
COPD/Pulmonary fibrosis	Muscularization of arterioles	Hypoxia rat/mouse
Obstructive sleep apnea		
**Group 4: Pulmonary hypertension due to chronic thrombotic and/or**		
**embolic disease**		
Pulmonary emboli	Thrombi or embolism	Vena cava ligation
Other clotting disorders	Recanalized organized thrombi	
**Group 5: Pulmonary hypertension triggered by other health conditions**		
Heterogeneous	Heterogeneous	

### Pathology of PH

Although the exact causes of PH remain to be determined, study findings indicate that it results from a combination of sustained pulmonary vasoconstriction and pulmonary vascular remodeling (Stenmark and McMurtry, 2005; Stenmark et al., 2006). Pulmonary vasoconstriction is the major contributor to the early phase of the disease; pulmonary vascular structural remodeling becomes progressively more dominant and important over time (Shimoda and Laurie, 2013). Hypoxic pulmonary vasoconstriction is a reflex contraction of vascular smooth muscle in the pulmonary circulation to optimize lung blood flow from low ventilated areas to well-oxygenated areas, and thereby optimize gas exchange and oxygen delivery (Moudgil et al., 2005; Dunham-Snary et al., 2017; Tarry and Powell, 2017). Unlike the systemic circulation, which dilates in the presence of hypoxia, pulmonary arteries constrict in response to alveolar hypoxia (Detar, 1980; Waypa and Schumacker, 2010). Hypoxic pulmonary vasoconstriction is an important homeostatic mechanism used to match regional perfusion and ventilation in the lung (Dunham-Snary et al., 2017; Tarry and Powell, 2017).

Sustained pulmonary vasoconstriction initiates pulmonary vascular structural changes. These changes are characterized by thickening of the intimal and/or medial layers of muscular vessels, which results in concentric pulmonary vascular remodeling (Heath and Edwards, 1958; Tuder, 2017). In human beings, pulmonary vascular remodeling is attributed to lesions that mainly occur in distal pre-capillary arteries, ranging in diameter from 500 to 700 μm. Remodeling involves a change in the maximal lumen diameter (interior and exterior) and accumulation of different vascular cell types in the pulmonary arterial wall (pulmonary artery ECs, VSMCs, and fibroblasts). Pulmonary endothelial dysfunction is the key trigger that drives PH development. It is characterized by either impairment of endothelial-dependent vasodilatation, reduced anticoagulant properties, ROS production, or active EC metabolic changes (Budhiraja et al., 2004; Attinà et al., 2005; Klinger et al., 2013; Ranchoux et al., 2018). Various stimuli (e.g., hypoxia, smoking, disturbed blood flow, and oxidative stress) can lead to endothelial dysfunction (Dummer et al., 2018; Ranchoux et al., 2018). In PH, progressive accumulation of resident VSMCs in pulmonary arteries contributes to expansion of the tunica media. Accumulating evidence also supports involvement of increased VSMC proliferation and inhibition of apoptosis in pulmonary vascular medial layer thickening (Tuder et al., 2007; Lyle et al., 2017; Humbert et al., 2019). Better understanding of the molecular mechanisms underlying pulmonary endothelial dysfunction and VSMC adaptation will greatly enhance our understanding of the pathogenesis of PH, which may help identify new therapeutic strategies. Other promising targets (e.g., fibroblast cell activation and immune system dysregulation) have also been identified as contributing to the pathogenesis of PH (Li M. et al., 2011; Rabinovitch et al., 2014; Plecitá-Hlavatá et al., 2016; Nicolls and Voelkel, 2017).

### Animal Models of PH

A variety of pre-clinical PH animal models are available to study this complex disease of diverse etiologies and histopathological features. Each model has its own hemodynamic and microanatomic histological characteristics (Table 1). The chronic hypoxia rat/mouse model is the one most widely used to study PH. Exposure of rats/mice to hypoxia causes increased mPAP, pulmonary vasoconstriction, and vascular medial hypertrophy that mimic the pathological features of human PH. However, right ventricular failure is absent (Zhao, 2010; Ryan et al., 2013). Monocrotaline (MCT) is a toxic alkaloid that causes a widespread pneumotoxicity and endothelial injury (Kay et al., 1967; Wilson et al., 1989). A single dose of MCT (60 mg/Kg) is sufficient to induce PH in rats by modulating two key pathological features of human PH, pulmonary vascular remodeling and right ventricular failure (Schoental and Head, 1955; Jasmin et al., 2001; Dumitrascu et al., 2008; Gomez-Arroyo et al., 2012). Sugen 5416 is a vascular endothelial growth factor receptor 2 (VEGFR2) inhibitor. Sugen 5416/hypoxia (Su/Hx) induces severe PH in both rats and mice that is characterized by pulmonary angioobliteration and right ventricular failure (Taraseviciene-Stewart et al., 2001; Sakao and Tatsumi, 2011). These three PH animal models are well-recognized models of Group 1 PH and Group 3 PH (Ryan et al., 2013; Colvin and Yeager, 2014; Sztuka and Jasiñska-Stroschein, 2017). Lai et al. (2016) developed a two-hit model of PH associated with heart failure with preserved ejection fraction (PH-HFpEF). It includes giving a single injection of SU5416 to obese ZSF1 rats. The SU5416/obese ZSF1 rats develop PH that includes a preserved ejection fraction and right and left ventricular hypertrophy. PH-HFpEF develops as a more advanced corollary of PH and diastolic HF, leading to more severe symptoms than those with HFpEF and suffers significant exercise intolerance, frequent hospitalization, and reduced survival (Thenappan et al., 2011; Hoeper et al., 2016).

## AMPK and Pulmonary Hypertension

### Role of AMPK in the Predisposition and Development of PH

Researches have revealed the role of AMPK in hypoxic pulmonary vasoconstriction and pulmonary vascular remodeling. Two clinical trials (NCT01884051 and NCT03629340) focusing on PAH treatment with metformin are in progress. However, in various animal models, AMPK has contradictory effects on PH, as both inhibition and activation of AMPK are protective for the development of PH. These seemingly opposing results can be partly explained by the different effects of AMPK signaling in pulmonary vasoconstriction and pulmonary vascular remodeling.

#### Role of AMPK in Hypoxic Pulmonary Vasoconstriction

Until 1871, it was universally believed that the pulmonary vessels did not respond to a vasomotor system. However, Brown-Séquard (1871) published some results indicating that such a system exists. Subsequently, Bradford and Dean (1894) reported that asphyxia causes PH. von Euler and von Liljestrand (1946) reported that acute hypoxia promotes pulmonary vasoconstriction to increase pulmonary arterial pressure. This study (von Euler and von Liljestrand, 1946) launched the current era of study of hypoxia and pulmonary vasoconstriction.

Hypoxic pulmonary vasoconstriction is an important homeostatic physiological mechanism that optimizes ventilation/perfusion matching, gas exchange, and systemic oxygen delivery. In response to alveolar hypoxia, intrapulmonary arteries constrict to divert blood to better-oxygenated lung segments (Bradford and Dean, 1894; von Euler and von Liljestrand, 1946; McMurtry et al., 1976; Madden et al., 1985; Sylvester et al., 2012; Dunham-Snary et al., 2017). Hypoxic pulmonary vasoconstriction relies on a group of specialized pulmonary VSMCs, which are located in pulmonary arterial segments stripped of the tunica intima and tunica media, but not in similar segments of pulmonary veins or systemic arteries (Bergofsky et al., 1967; Murray et al., 1990a, b; Madden et al., 1992; Weir and Archer, 1995). Hypoxic pulmonary vasoconstriction is triggered by mitochondrial redox signaling that involves voltage-gated potassium channels (Kv) and calcium channels (Weir and Archer, 1995). Hypoxia inhibits Kv channels in pulmonary VSMCs, causing membrane depolarization and opening of voltage-gated calcium channels to initiate Ca^2+^-mediated pulmonary vasoconstriction (Weir and Archer, 1995; Archer and Michelakis, 2002; Sommer et al., 2008; Dunham-Snary et al., 2017).

**FIGURE 2:**
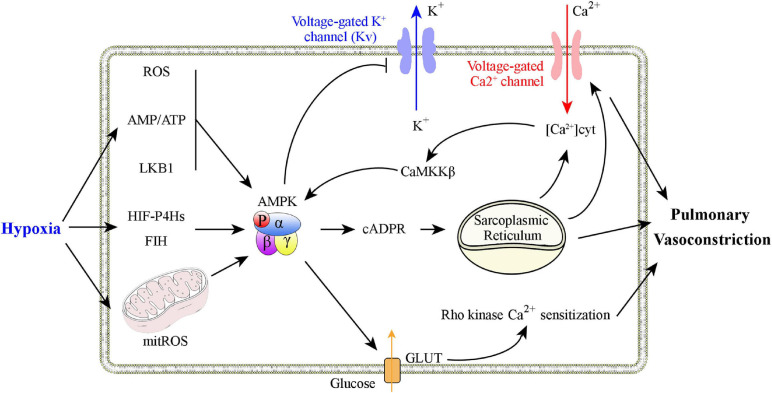
Graphical summary of possible mechanisms that explain how AMPK activation is involved in hypoxic pulmonary vasoconstriction. Physiological hypoxia results in increases in the AMP/ATP ratio, ROS production, and in LKB1 activation to promote AMPK Thr 172 phosphorylation. AMPK activation increases Ca^2+^ levels in pulmonary VSMCs to promote constriction through, (1) inhibition of voltage-dependent K^+^ channels, (2) activation of voltage-operated Ca^2+^ channels, and (3) activation of Ca^2+^-sensing sarcoplasmic reticulum.

Adenosine monophosphate-activated protein kinase has a critical role in hypoxic pulmonary vasoconstriction by linking the oxygen sensor to its effectors (Figure 2). Evans et al. (2005, 2006) and Evans (2006) found that physiological hypoxia increases the AMP/ATP ratio in pulmonary VSMCs, followed by increased AMPK activity and phosphorylation of a classical AMPK substrate, acetyl CoA carboxylase (a well-validated marker for AMPK activation). This process is likely to be mediated by binding of AMP to the AMPK γ subunit, which triggers activation of the kinase by, (1) promoting AMPK Thr 172 phosphorylation *via* allosteric regulation (Scott et al., 2002; Kemp, 2004; Oakhill et al., 2010), (2) inhibiting AMPK Thr 172 dephosphorylating (Davies et al., 1995), and (3) facilitating phosphorylation of Thr 172 by the upstream kinase LKB1 (Hawley et al., 2003; Woods et al., 2003; Shaw et al., 2004, 2005). Additional studies found that AMPK activation evokes a slow, sustained, and reversible increase in Ca^2+^ influx *via* cyclic adenosine diphosphate-ribose (cADPR)-dependent mobilization of sarcoplasmic reticulum stores in pulmonary VSMCs and the consequent induction of constriction of pulmonary artery rings (Evans et al., 2005). Consistent with these findings, two different AMPK activators, AICAR and phenformin, evoke intracellular Ca^2+^ influx and reversible constriction of the pulmonary artery rings. The characteristics of this process are strikingly similar to those of hypoxic pulmonary vasoconstriction (Evans et al., 2005). The hypoxia-associated pulmonary vasoconstriction and Ca^2+^ influx is inhibited by the non-selective AMPK antagonist, compound C, upon inhibition of the sarcoplasmic reticulum store-refilling current (Robertson et al., 2008). When hypoxia occurs, AMPK can directly phosphorylate voltage-gated potassium channels (Kv1.5 channels), followed by inhibition of K^+^ currents in pulmonary VSMCs. The entry of voltage-dependent Ca^2+^ to initiate the hypoxia-related pulmonary vasoconstriction is thus activated (Moral-Sanz et al., 2016). Downregulation of Kv1.5 expression and activity is also a hallmark of PH (Yuan et al., 1998; Lv et al., 2013). Strong support for this mechanism results from *in vivo* studies performed by Moral-Sanz et al. (2018), who found a key *in vivo* role of AMPK in hypoxic pulmonary vasoconstriction using a combination of AMPK isoform deletion strategies and spectral Doppler ultrasound. Under conditions of mild hypoxia (8% O_2_), deletion of AMPK α1, but not AMPK α2, in smooth muscle cells block induction of hypoxia-related pulmonary vasoconstriction. When conditions of severe hypoxia (5% O_2_) are present, either AMPK α1 or AMPK α2 deletion attenuates hypoxia-related pulmonary vasoconstriction (Moral-Sanz et al., 2018). The findings that SNPs in the *Prkaa1* gene have been identified in populations that live at high altitudes and who have attenuated hypoxic pulmonary vasoconstriction are consistent with these results (Penaloza and Arias-Stella, 2007; Bigham et al., 2014). In summary, a growing body of evidence supports the hypothesis that AMPK activation is a primary mediator of hypoxic pulmonary vasoconstriction.

#### Role of AMPK in Pulmonary Vasculature Remodeling

**FIGURE 3:**
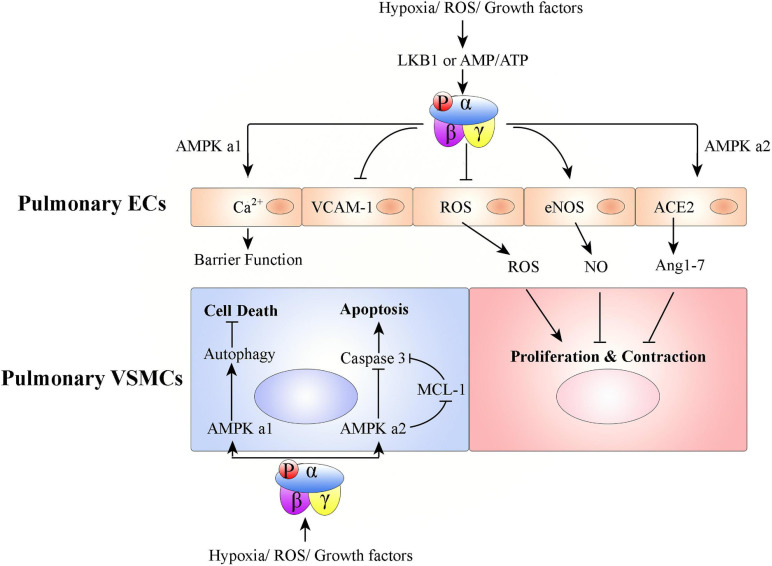
Graphical summary of possible mechanisms associated with how AMPK activation affects pulmonary vascular remodeling. Hypoxia/ROS/growth factors activate AMPK in ECs or VSMCs to promote or inhibit PH development. In pulmonary ECs, AMPK activation protects PH: (1) AMPK α1 activation in pulmonary ECs promotes Ca^2+^ influx to maintain endothelial barrier function, (2) pulmonary endothelial AMPK inhibits VCAM-1 expression to antagonize inflammatory cell infiltration, (3) AMPK activation in pulmonary ECs inhibits ROS production, promotes eNOS-NO bioactivity, and stabilizes ACE2 to increase Ang 1–7 expression. Pulmonary VSMC proliferation and contraction are thus regulated. In pulmonary VSMCs, AMPK α1 activation promotes autophagy-mediated cell survival; AMPK α2 activation inhibits cell apoptosis.

In PH, pulmonary arteries and veins undergo structure changes. This pulmonary vascular remodeling is characterized by proliferation of pulmonary ECs and VSMCs. AMPK has a key role in the pathogenesis of pulmonary vasculature remodeling (Figure 3).

##### AMPK in Endothelial Cells and PH

Both AMPK subunits (AMPK α1 and AMPK α2) are expressed in pulmonary ECs. However, AMPK α1 is mainly expressed in capillary-derived pulmonary microvascular ECs and AMPK α2 is mainly expressed in conduit-derived pulmonary artery ECs (Creighton et al., 2011). Pulmonary endothelial AMPK is down-regulated in pulmonary hypertensive patients and hypoxia-induced PH animals, compared with that from healthy donors or non-PH animals, respectively (Teng et al., 2013; Afolayan et al., 2016; Omura et al., 2016; Rana et al., 2020). EC-specific AMPK knockout mice (EC-AMPK^–/–^) (Omura et al., 2016) and EC-specific AMPK α2 knockout mice (EC-AMPK α2^–/–^) (Zhang et al., 2018) have accelerated development of PH under conditions of hypoxia. Endothelial AMPK exerts protective effects against PH via, (1) paracrine growth mechanism (e.g., PDGF-BB and FGF-2) mediation of the interaction between pulmonary artery ECs and pulmonary VSMCs (Omura et al., 2016), (2) positive regulation of nitric oxide (NO) production *via* endothelial NO synthase (eNOS) phosphorylation (Zhang et al., 2006; Chen et al., 2009; Afolayan et al., 2016), (3) stabilization of angiotensin-converting enzyme 2 (ACE2) to increase angiotensin 1–7 (Ang 1–7) expression and eNOS-derived NO bioavailability (Zhang et al., 2006), (4) Ca^2+^ influx in pulmonary microvascular ECs, which promotes endothelial barrier function (Creighton et al., 2011), (5) notch ligand promotion of angiogenesis (200), and (6) alleviation of EC apoptosis (Ido et al., 2002; Nagata et al., 2009; Enkhjargal et al., 2014).

##### AMPK in Vascular Smooth Muscle Cells and PH

During PH development, the remodeling process universally involves medial thickening driven by VSMC proliferation/hypertrophy and deposition of extracellular matrix within the tunica media of pulmonary arteries (Lyle et al., 2017). Pulmonary VSMCs express both the AMPK α1 and AMPK α2 subunits of AMPK (Creighton et al., 2011; Ibe et al., 2013). However, AMPK α1 is the predominant subunit in pulmonary VSMCs and contributes up to 80% of total AMPK activity (Evans et al., 2005; Xue et al., 2017). AMPK α1 catalytic activity is much higher in VSMCs from small pulmonary arteries than in those from the main pulmonary arteries (Evans et al., 2005). Unlike AMPK in pulmonary ECs, studies of AMPK in pulmonary VSMCs have found contradictory results. Some studies found that phosphorylated AMPK is increased, while total AMPK levels remain the same, in pulmonary VSMCs from pulmonary hypertensive patients and hypoxia-induced PH mice, compared with those from healthy donors or non-PH mice, respectively (Krymskaya et al., 2011; Ibe et al., 2013). Mechanistically, hypoxia-activated AMPK promotes pulmonary VSMC survival, but AMPK activity pharmacologically inhibited by either compound C or 9-β-d-arabinofuranosyl adenine (Ara-a) abrogates hypoxia-induced pulmonary VSMC proliferation and PH (Ibe et al., 2013; Xue et al., 2017). Ibe et al. (2013) found that although suppression of either AMPK α1 or α2 in pulmonary VSMCs leads to increased cell death, AMPK α1 and AMPK α2 have differential roles. Activation of AMPK α1 stimulates autophagy and promotes pulmonary VSMC survival; activation of AMPK α2 regulates myeloid cell leukemia sequence 1 (MCL-1) to prevent apoptosis (Ibe et al., 2013). In contrast to these results, another series of studies found that phosphorylated AMPK is decreased in pulmonary VSMCs from patients with PH and from mice with hypoxia-induced PH, compared with those from healthy donors or non-PH mice, respectively (Goncharov et al., 2014). AMPK inhibition promotes pulmonary VSMC proliferation and survival, but AMPK pharmacologically activated by metformin or AICAR inhibits hypoxia-induced pulmonary VSMC proliferation and chronic PH (Agard et al., 2009; Goncharov et al., 2014; Wu et al., 2014; Ke et al., 2016; Song et al., 2016; Gui et al., 2017; Liu et al., 2019).

Seemingly contradictory results should be interpreted with caution. In the study that found elevated AMPK phosphorylation in PH and AMPK inhibition-attenuated PH (Krymskaya et al., 2011; Ibe et al., 2013), the researchers used pulmonary VSMCs isolated from large-diameter arteries located in a segment of pulmonary arteries just proximal to where lung entry occurs (diameter ≥ 0.8 mm). In the study that found AMPK reduction in PH and PH mitigation by AMPK activation (Goncharov et al., 2014), pulmonary VSMCs isolated from small-diameter arteries located in distal pulmonary artery segments (type III, diameter ≤ 0.1 mm) were used. Therefore, these discrepancies may be due to different functions of AMPK or different AMPK isoforms, or both, in pulmonary arteries with different diameters. Hypoxic pulmonary vasoconstriction is more vigorous in small pulmonary arteries (Dawson et al., 1977; Grimm et al., 1978; Sylvester et al., 2012), where the AMPK α1 catalytic subunit is predominantly expressed (Evans et al., 2005). The non-selective nature of AMPK activators or inhibitors may be another factor that contributes to these apparent inconsistencies.

### AMPK and Pulmonary Hypertension Treatment

#### AMPK Inhibition Is Preventive for Development of Pulmonary Hypertension

Ibe et al. (2013) found that inhibition of AMPK by compound C prevents development of hypoxia-induced PH. When mice treated with compound C one day before hypoxia exposure (10% oxygen for 3 weeks), compound C prevents hypoxia-induced PH, pulmonary arterial wall thickening, and right ventricular hypertrophy. The activation of AMPK α1 stimulates autophagy, promoting pulmonary VSMCs survival, whereas the activation of AMPK α2 increases the expression of myeloid cell leukemia sequence 1 (MCL-1), inhibiting pulmonary VSMCs apoptosis (Ibe et al., 2013). Consistent with these results, Robertson et al. (2008) and Evans et al. (2009) pre-incubated intrapulmonary arteries (3rd and 4th order branches of the pulmonary arterial tree, 0.2–0.5 mm internal diameter) with compound C (40 mM). Compound C reversed/inhibited hypoxic pulmonary vasoconstriction in a concentration-dependent manner. Functionally, AMPK phosphorylates voltage-gated K^+^ channel (Kv2.1) and thereby confers a leftward shift in both the activation and inactivation curves of Kv2.1, which precipitates an increase in the intracellular Ca^2+^ concentration (Evans et al., 2009).

#### AMPK Activation Is Preventive for Development of Pulmonary Hypertension

A significant body of evidence suggests that AMPK activation is preventive for development of PH. Metformin, the first-line medication for treatment of type 2 diabetes and the canonical AMPK activator, demonstrates therapeutic efficacy on PH in animal models. AMPK activation by metformin prevents MCT-induced PH in rats (Agard et al., 2009; Li et al., 2016; Zhai et al., 2018; Sun et al., 2019; Yoshida et al., 2020). In these experimental models, rats were injected with one dose of MCT (60 mg/kg) to induce PH. Metformin (100–150 mg/kg/day, drinking water or intraperitoneal injection, 21–30 days) treatment significantly reduced right ventricular systolic pressure and pulmonary vascular remodeling in rats with MCT-induced PH (Agard et al., 2009; Li et al., 2016; Zhai et al., 2018; Sun et al., 2019; Yoshida et al., 2020). Consistent with these results, metformin has protective effects on hypoxia-induced PH in mice and rats (Huang et al., 2014; Omura et al., 2016; Liu et al., 2019), and a more pronounced PH with angioobliterative lesions in Sugen 5416/hypoxia (SuHx) mice/rats (Dean et al., 2016; Zhang et al., 2018) and SU5416/Obese ZSF1 rats (Lai et al., 2016; Wang et al., 2020). The AMPK activator, AICAR, also prevents PH development in rats with hypoxia-induced PH (Huang et al., 2014; Chen et al., 2016).

In contrast to the results that metformin has protective effects, other researchers reported that their findings did not support the efficacy of metformin in PAH therapy (Goncharov et al., 2018). Goncharov et al. (2018) findings suggested that metformin treatment may be preferentially beneficial for PH with heart failure with preserved ejection fraction (PH-HFpEF, group 2 PH), but that it has limited efficacy for PAH. They induced PAH in a male C57BL/6J mouse model using a 3-week exposure to SuHx. They then gave metformin (100 mg/kg/day, 14 days) in drinking water for 2-weeks post hypoxia exposure. Goncharov et al. found no changes in right ventricular systolic pressure, right ventricular hypertrophy, or pulmonary vascular remodeling in the metformin-treated SuHx mice. They also evaluated the preventive effects of metformin and AICAR in PAH. In these animal models, metformin (300 mg/kg/day, drinking water, 42 days) or AICAR (500 mg/kg/day, intraperitoneal, 42 days) were administrated 1 day before SuHx exposure. They found no changes in right ventricular systolic pressure, right ventricular hypertrophy, or pulmonary vascular remodeling in either the metformin- or AICAR-treated SuHx rats. However, they did not measure phosphorylation levels of AMPK or downstream AMPK pathways. Metformin-induced AMPK activation requires full activation of an upstream kinase (e.g., LKB1), especially at low doses (Choi et al., 2001; Emerling et al., 2009; Zmijewski et al., 2010; Hinchy et al., 2018). Therefore, it is unknown whether the lack of metformin efficacy for PH treatment was associated with AMPK activation.

Different characteristics that likely contribute to apparently contradictory results are presented in Table 2. In Dean et al. (2016), AMPK activation by metformin (100 mg/kg/day, oral gavage, 21 days) seems to reverse the PH phenotype induced by SuHx in female rats, in contrast to the findings of Goncharov et al. (2018) that was performed using male rats. Thus, a sex difference might affect the response to metformin treatment of PH. Effects of this difference have been described for other diseases (e.g., obesity, aging, and spontaneous tumorigenesis) (Anisimov et al., 2010; Quan et al., 2016; Park et al., 2017; Bramante et al., 2021).

**TABLE 2:** Pulmonary hypertension (PH) animal experiments of AMPK.

Therapy	Dose (mg/kg/day)	Route	Time (day)	PH models	Sex	References
**AMPK activator with beneficial effects on PH**						
Metformin	100–150	p.o.	21–28	MCT rat	M	Zhai et al., 2018; Yoshida et al., 2020
Metformin	100–150	i.p.	21–28	MCT rat	M	Agard et al., 2009; Li et al., 2016
Metformin	100	i.p.	30	MCT rat		Sun et al., 2019
Metformin	100	i.p.	21	Hypoxia rat		Liu et al., 2019
Metformin	100	p.o.	21	Su/Hx rat	F	Dean et al., 2016
Metformin	300	p.o.	31–28	Obese ZSF1 rat	M	Lai et al., 2016
Metformin	300	p.o.	98	SU5416/Obese ZSF1 rat	F	Wang et al., 2020
Metformin	150	i.p.	14	Su/Hx mouse	M	Zhang et al., 2018
Metformin	100	p.o.	21	Hypoxia mouse	M	Omura et al., 2016
AICAR	1?	i.p.	28	Hypoxia rat	M	Huang et al., 2014; Chen et al., 2016
**AMPK antagonist with beneficial effects on PH**						
Compound C	20	i.p.	21	Hypoxia mouse	M	Ibe et al., 2013
**AMPK activators without effects on PH**						
Metformin	100	p.o.	14	SuHx mouse	M	Goncharov et al., 2018
Metformin	300	p.o.	42	SuHx rat	M	Goncharov et al., 2018
AICAR	500	p.o.	42	SuHx rat	M	Goncharov et al., 2018

*p.o., oral administration (*per os*); i.p., Intraperitoneal injection.*

### Clinical Trials of Pulmonary Hypertension Treatment Using Metformin

There has been significant interest in the use of metformin for PH treatment. Liao et al. (2018, 2019) found that a combination therapy using metformin (500 mg, twice daily, 3 months) and bosentan (endothelin receptor antagonist) improves 6-min walk distance and right heart hemodynamics, decreases serum pro-brain natriuretic peptide (pro-BNP) levels, and ameliorates pulmonary vasoconstriction in patients with PH associated with congenital heart defects. Two phase I/II clinical trials of metformin for PH treatment are in progress (clinicaltrials.gov, NCT01884051 and NCT03629340). Results to date indicate good tolerability and potential clinical efficacy for improvement in right ventricular function in patients with PH who receive metformin therapy (2 g/day, 8 weeks) (Brittain et al., 2020). However, metformin use did not change the 6-min walk distance in those patients (Brittain et al., 2020). Although not yet complete, this clinical study provides new insights into the potential benefits of metformin use on right ventricular failure in patients with PH and indicates the need for more studies of the use of metformin therapeutic intervention in patients with PH and PH-HFpEF.

## Conclusion and Perspectives

In this review, we discussed some seemingly contradictory study results for AMPK and PH development. AMPK has a key role in PH, either during the early process of hypoxic pulmonary vasoconstriction or later during pulmonary vasculature remodeling, or both. However, whether AMPK activation or inhibition is protective against PH remains unclear: (1) AMPK activation triggers hypoxia-induced pulmonary artery constriction. AMPK activator use (e.g., AICAR and Ara-a) prevents hypoxia-induced pulmonary artery constriction and PH. (2) EC-specific deletion of AMPK exaggerates hypoxia-induced PH *in vivo*. This result indicates endothelial AMPK has a protective role during PH development. (3) VSMCs from large pulmonary arteries with AMPK activation have accelerated proliferation and inhibited apoptosis. VSMCs from distal small pulmonary arteries with AMPK inhibition have similar potential. (4) Some animal studies found that the AMPK activators, AICAR and metformin, have beneficial effects on PH treatment. Other study findings suggest that metformin therapy for PH may be limited to use for PH-HFpEF. AMPK activation might have less pronounced pulmonary vascular effects than right ventricular effects, as much evidence has been published suggesting that AMPK activation exerts a protective effect in cardiac dysfunction, ischemic heart, heat failure, and cardiac hypertrophy (Russell et al., 2004; Miller et al., 2008; Ma et al., 2010; Kim et al., 2011; Morrison et al., 2011). (5) Sex differences in the response to metformin used for PH treatment may affect outcomes.

In conclusion, studies found seemingly contradictory results for the relationship between AMPK and PH. In one series of studies, inhibition of AMPK resulted in attenuated hypoxic pulmonary vasoconstriction and pulmonary VSMC proliferation. In another series of studies, activation of AMPK resulted in improved EC function, VSMC apoptosis, and decreased pulmonary vasculature tone. Given that AMPK a1 and AMPK a2 have different expression patterns and different functions in pulmonary arteries of different sizes, the role of AMPK in PH should be studied using a cell-specific and pathological process-specific approach. Studies involving genetically- and specifically-modified AMPK α1 and α2 subunits are needed to clarify their specific roles in PH pathogenesis and treatment.

## Author Contributions

QZ drafted the manuscript and figures. PS and M-HZ revised the manuscript. All authors contributed to the article and approved the submitted version.

## Conflict of Interest

The authors declare that the research was conducted in the absence of any commercial or financial relationships that could be construed as a potential conflict of interest.

## References

[B1] Afolayan A. J. Eis A. Alexander M. Michalkiewicz T. Teng R.-J. Lakshminrusimha S. (2016). Decreased endothelial nitric oxide synthase expression and function contribute to impaired mitochondrial biogenesis and oxidative stress in fetal lambs with persistent pulmonary hypertension. *Am. J. Physiol. Lung Cell. Mol. Physiol.* 310 L40–L49. 26519208 10.1152/ajplung.00392.2014 PMC4698434

[B2] Agard C. Rolli-Derkinderen M. Dumas-de-La-Roque E. Rio M. Sagan C. Savineau J. P. (2009). Protective role of the antidiabetic drug metformin against chronic experimental pulmonary hypertension. *Br. J. Pharmacol.* 158 1285–1294. 10.1111/j.1476-5381.2009.00445.x 19814724 PMC2782337

[B3] Ahmad F. Arad M. Musi N. He H. Wolf C. Branco D. (2005). Increased alpha2 subunit-associated AMPK activity and PRKAG2 cardiomyopathy. *Circulation* 112 3140–3148. 10.1161/circulationaha.105.550806 16275868

[B4] Anisimov V. N. Piskunova T. S. Popovich I. G. Zabezhinski M. A. Tyndyk M. L. Egormin P. A. (2010). Gender differences in metformin effect on aging, life span and spontaneous tumorigenesis in 129/Sv mice. *Aging (Albany N. Y.)* 2 945–958. 10.18632/aging.100245 21164223 PMC3034183

[B5] Arad M. Seidman C. E. Seidman J. G. (2007). AMP-activated protein kinase in the heart. *Circulat. Res.* 100 474–488. 17332438 10.1161/01.RES.0000258446.23525.37

[B6] Archer S. Michelakis E. (2002). The mechanism(s) of hypoxic pulmonary vasoconstriction: potassium channels redox O2 sensors, and controversies. *Physiology* 17 131–137. 10.1152/nips.01388.2002 12136039

[B7] Attinà T. Camidge R. Newby D. E. Webb D. J. (2005). Endothelin antagonism in pulmonary hypertension, heart failure, and beyond. *Heart* 91 825–831. 10.1136/hrt.2004.053991 15894792 PMC1768938

[B8] Bando H. Atsumi T. Nishio T. Niwa H. Mishima S. Shimizu C. (2005). Phosphorylation of the 6-phosphofructo-2-kinase/fructose 2,6-bisphosphatase/PFKFB3 family of glycolytic regulators in human cancer. *Clin. Cancer Res.* 11 5784–5792. 10.1158/1078-0432.ccr-05-0149 16115917

[B9] Bergeron R. Ren J. M. Cadman K. S. Moore I. K. Perret P. Pypaert M. (2001). Chronic activation of AMP kinase results in NRF-1 activation and mitochondrial biogenesis. *Am. J. Physiol. Endocrinol. Metabol.* 281 E1340–E1346. 10.1152/ajpendo.2001.281.6.E1340 11701451

[B10] Bergofsky E. H. Holtzman S. Study A. (1967). A study of the mechanisms involved in the pulmonary arterial pressor response to hypoxia. *Circulat. Res.* 20 506–519. 10.1161/01.res.20.5.506 6057684

[B11] Bigham A. W. Julian C. G. Wilson M. J. Vargas E. Browne V. A. Shriver M. D. (2014). Maternal PRKAA1 and EDNRA genotypes are associated with birth weight, and PRKAA1 with uterine artery diameter and metabolic homeostasis at high altitude. *Physiol. Genom.* 46 687–697. 10.1152/physiolgenomics.00063.2014 25225183 PMC4166715

[B12] Bradford J. R. Dean H. P. (1894). The pulmonary circulation. *J. Physiol.* 16 34–158.25. 10.1113/jphysiol.1894.sp000493 PMC1514499 16992161

[B13] Bramante C. T. Ingraham N. E. Murray T. A. Marmor S. Hovertsen S. Gronski J. (2021). Metformin and risk of mortality in patients hospitalised with COVID-19: a retrospective cohort analysis. *Lancet Health. Longev.* 2 e34–e41. 10.1016/S2666-7568(20)30033-7 PMC7832552 33521772

[B14] Brittain E. L. Niswender K. Agrawal V. Chen X. Fan R. Pugh M. E. (2020). Mechanistic phase II clinical trial of metformin in pulmonary arterial hypertension. *J. Am. Heart Associat.* 9:e018349. 10.1161/JAHA.120.018349 PMC7763730 33167773

[B15] Brown-Séquard C. E. (1871). On the production of hæmorrhage, anæmia, śdema, and emphysema in the lungs by injuries to the base of the brain. *Lancet* 97:6. 10.1016/s0140-6736(02)77750-7 24679462

[B16] Budhiraja R. Tuder R. M. Hassoun P. M. (2004). Endothelial dysfunction in pulmonary hypertension. *Circulation* 109 159–165. 14734504 10.1161/01.CIR.0000102381.57477.50

[B17] Bungard D. Fuerth B. J. Zeng P.-Y. Faubert B. Maas N. L. Viollet B. (2010). Signaling kinase AMPK activates stress-promoted transcription via histone H2B phosphorylation. *Science* 329 1201–1205. 10.1126/science.1191241 20647423 PMC3922052

[B18] Carling D. Zammit V. A. Hardie D. G. (1987). A common bicyclic protein kinase cascade inactivates the regulatory enzymes of fatty acid and cholesterol biosynthesis. *FEBS Lett.* 223 217–222. 10.1016/0014-5793(87)80292-2 2889619

[B19] Chandra S. M. Razavi H. Kim J. Agrawal R. Kundu R. K. de Jesus Perez V. (2011). Disruption of the apelin-APJ system worsens hypoxia-induced pulmonary hypertension. *Arterioscler. Thrombos. Vascul. Biol.* 31 814–820. 10.1161/atvbaha.110.219980 21233449 PMC3113525

[B20] Chen L. Xin F.-J. Wang J. Hu J. Zhang Y.-Y. Wan S. (2013). Conserved regulatory elements in AMPK. *Nature* 498 E8–E10. 23765502 10.1038/nature12189

[B21] Chen M. Cai H. Yu C. Wu P. Fu Y. Xu X. (2016). Salidroside exerts protective effects against chronic hypoxia-induced pulmonary arterial hypertension via AMPKα1-dependent pathways. *Am. J. Transl. Res.* 8 12–27. 27069536 PMC4759412

[B22] Chen Z. Peng I. C. Sun W. Su M. I. Hsu P. H. Fu Y. (2009). AMP-activated protein kinase functionally phosphorylates endothelial nitric oxide synthase Ser633. *Circulat. Res.* 104 496–505. 10.1161/circresaha.108.187567 19131647 PMC2761102

[B23] Cheung P. C. Salt I. P. Davies S. P. Hardie D. G. Carling D. (2000). Characterization of AMP-activated protein kinase gamma-subunit isoforms and their role in AMP binding. *Biochem. J.* 346(Pt 3) 659–669. 10.1042/0264-6021:3460659 10698692 PMC1220898

[B24] Choi S.-L. Kim S.-J. Lee K.-T. Kim J. Mu J. Birnbaum M. J. (2001). The regulation of AMP-activated protein kinase by H2O2. *Biochem. Biophys. Res. Commun.* 287 92–97. 11549258 10.1006/bbrc.2001.5544

[B25] Colvin K. L. Yeager M. E. (2014). Animal models of pulmonary hypertension: matching disease mechanisms to etiology of the human disease. *J. Pulmon. Respirat. Med.* 4:198. 10.4172/2161-105X.1000198 PMC4334132 25705569

[B26] Creighton J. Jian M. Sayner S. Alexeyev M. Insel P. A. (2011). Adenosine monophosphate-activated kinase alpha1 promotes endothelial barrier repair. *FASEB J.* 25 3356–3365. 10.1096/fj.10-179218 21680893 PMC3177581

[B27] Davies S. P. Helps N. R. Cohen P. T. W. Hardie D. G. (1995). 5′-AMP inhibits dephosphorylation, as well as promoting phosphorylation, of the AMP-activated protein kinase. Studies using bacterially expressed human protein phosphatase-2Cα and native bovine protein phosphatase-2Ac. *FEBS Lett.* 377 421–425. 10.1016/0014-5793(95)01368-7 8549768

[B28] Dawson C. A. Grimm D. J. Linehan J. H. (1977). Effects of lung inflation on longitudinal distribution of pulmonary vascular resistance. *J. Appl. Physiol.* 43 1089–1092. 10.1152/jappl.1977.43.6.1089 606694

[B29] Day E. A. Ford R. J. Steinberg G. R. (2017). AMPK as a therapeutic target for treating metabolic diseases. *Trends Endocrinol. Metabol.* 28 545–560. 10.1016/j.tem.2017.05.004 28647324

[B30] de Theije C. C. Schols A. Lamers W. H. Neumann D. Köhler S. E. Langen R. C. J. (2018). Hypoxia impairs adaptation of skeletal muscle protein turnover- and AMPK signaling during fasting-induced muscle atrophy. *PLoS One* 13:e0203630. 10.1371/journal.pone.0203630 30212583 PMC6136752

[B31] Dean A. Nilsen M. Loughlin L. Salt I. P. MacLean M. R. (2016). Metformin reverses development of pulmonary hypertension via aromatase inhibition. *Hypertension* 68 446–454. 10.1161/hypertensionaha.116.07353 27296990

[B32] Dengler F. Gäbel G. (2019). The fast lane of hypoxic adaptation: glucose transport is modulated via a HIF-hydroxylase-AMPK-axis in jejunum epithelium. *Int. J. Mol. Sci.* 20:4993. 10.3390/ijms20204993 31601024 PMC6834319

[B33] Detar R. (1980). Mechanism of physiological hypoxia-induced depression of vascular smooth muscle contraction. *Am. J. Physiol.* 238 H761–H769. 6247924 10.1152/ajpheart.1980.238.6.H761

[B34] Ducommun S. Deak M. Sumpton D. Ford R. J. úñez Galindo A. N. Kussmann M. (2015). affinity and mass spectrometry proteomic approach for the discovery of cellular AMPK targets: identification of mitochondrial fission factor as a new AMPK substrate. *Cell. Signal.* 27 978–988. 10.1016/j.cellsig.2015.02.008 25683918

[B35] Dumitrascu R. Koebrich S. Dony E. Weissmann N. Savai R. Pullamsetti S. S. (2008). Characterization of a murine model of monocrotaline pyrrole-induced acute lung injury. *BMC Pulmon. Med.* 8:25. 10.1186/1471-2466-8-25 PMC2635347 19087359

[B36] Dummer A. Rol N. Szulcek R. Kurakula K. Pan X. Visser B. I. (2018). Endothelial dysfunction in pulmonary arterial hypertension: loss of cilia length regulation upon cytokine stimulation. *Pulmon. Circulat.* 8:2045894018764629. 10.1177/2045894018764629 PMC5858634 29480152

[B37] Dunham-Snary K. J. Wu D. Sykes E. A. Thakrar A. Parlow L. R. G. Mewburn J. D. (2017). Hypoxic pulmonary vasoconstriction: from molecular mechanisms to medicine. *Chest* 151 181–192. 27645688 10.1016/j.chest.2016.09.001 PMC5310129

[B38] Egan D. F. Shackelford D. B. Mihaylova M. M. Gelino S. Kohnz R. A. Mair W. (2011). of ULK1 (hATG1) by AMP-activated protein kinase connects energy sensing to mitophagy. *Science* 331 456–461. 10.1126/science.1196371 21205641 PMC3030664

[B39] Emerling B. M. Weinberg F. Snyder C. Burgess Z. Mutlu G. M. Viollet B. (2009). Hypoxic activation of AMPK is dependent on mitochondrial ROS but independent of an increase in AMP/ATP ratio. *Free Rad. Biol. Med.* 46 1386–1391. 10.1016/j.freeradbiomed.2009.02.019 19268526 PMC3326346

[B40] Emilio P. Mottillo E. M. Desjardins J. D. Crane B. K. Smith A. E. Green S. D. (2016). Lack of adipocyte AMPK exacerbates insulin resistance and hepatic steatosis through brown and beige adipose tissue function. *Cell Metab.* 24 118–129. 10.1016/j.cmet.2016.06.006 27411013 PMC5239668

[B41] Enkhjargal B. Godo S. Sawada A. Suvd N. Saito H. Noda K. (2014). Endothelial AMP-activated protein kinase regulates blood pressure and coronary flow responses through hyperpolarization mechanism in mice. *Arterioscl. Thromb. Vascul. Biol.* 34 1505–1513. 10.1161/atvbaha.114.303735 24855056

[B42] Evans A. M. (2006). AMP-activated protein kinase underpins hypoxic pulmonary vasoconstriction and carotid body excitation by hypoxia in mammals. *Exp. Physiol.* 91 821–827. 10.1113/expphysiol.2006.033514 16740641

[B43] Evans A. M. Hardie D. G. Peers C. Wyatt C. N. Viollet B. Kumar P. (2009). Ion channel regulation by AMPK. *Annal. N. Y. Acad. Sci.* 1177 89–100. 10.1111/j.1749-6632.2009.05041.x 19845611

[B44] Evans A. M. Mustard K. J. Wyatt C. N. Dipp M. Kinnear N. P. Hardie D. G. (2006). Does AMP-activated protein kinase couple inhibition of mitochondrial oxidative phosphorylation by hypoxia to pulmonary artery constriction? *Adv. Exp. Med. Biol.* 580 147–154; discussion 351–9. 16683711 10.1007/0-387-31311-7_22

[B45] Evans A. M. Mustard K. J. Wyatt C. N. Peers C. Dipp M. Kumar P. (2005). Does AMP-activated protein kinase couple inhibition of mitochondrial oxidative phosphorylation by hypoxia to calcium signaling in O_2_-sensing cells? *J. Biol. Chem.* 280 41504–41511. 10.1074/jbc.m510040200 16199527

[B46] Fogarty S. Hardie D. G. (2010). Development of protein kinase activators: AMPK as a target in metabolic disorders and cancer. *Biochim. Biophys. Acta (BBA) Prot. Proteom.* 1804 581–591. 10.1016/j.bbapap.2009.09.012 19778642

[B47] Fullerton M. D. Galic S. Marcinko K. Sikkema S. Pulinilkunnil T. Chen Z.-P. (2013). Single phosphorylation sites in Acc1 and Acc2 regulate lipid homeostasis and the insulin-sensitizing effects of metformin. *Nat. Med.* 19 1649–1654. 10.1038/nm.3372 24185692 PMC4965268

[B48] Galic S. Fullerton M. D. Schertzer J. D. Sikkema S. Marcinko K. Walkley C. R. (2011). Hematopoietic AMPK β1 reduces mouse adipose tissue macrophage inflammation and insulin resistance in obesity. *J. Clin. Investigat.* 121 4903–4915. 10.1172/jci58577 22080866 PMC3226000

[B49] Galiè N. Simonneau G. (2013). The fifth world symposium on pulmonary hypertension. *J. Am. Coll. Cardiol.* 62 D1–D3. 24355633 10.1016/j.jacc.2013.10.030

[B50] Galiè N. Hoeper M. M. Humbert M. Torbicki A. Vachiery J. L. Barbera J. A. (2009). Guidelines for the diagnosis and treatment of pulmonary hypertension. *Eur. Respirat. J.* 34 1219– 1263. 19749199 10.1183/09031936.00139009

[B51] Garcia-Roves P. M. Osler M. E. Holmström M. H. Zierath J. R. (2008). Gain-of-function R225Q mutation in AMP-activated protein Kinase γ3 subunit increases mitochondrial biogenesis in glycolytic skeletal muscle. *J. Biol. Chem.* 283 35724–35734. 10.1074/jbc.m805078200 18838377

[B52] Gomez-Arroyo J. G. Farkas L. Alhussaini A. A. Farkas D. Kraskauskas D. Voelkel N. F. (2012). The monocrotaline model of pulmonary hypertension in perspective. *Am. J. Physiol. Lung Cell. Mol. Physiol.* 302 L363–L369. 21964406 10.1152/ajplung.00212.2011

[B53] Goncharov D. A. Goncharova E. A. Tofovic S. P. Hu J. Baust J. J. Pena A. Z. (2018). Metformin therapy for pulmonary hypertension associated with heart failure with preserved ejection fraction versus pulmonary arterial hypertension. *Am. J. Respirat. Crit. Care Med.* 198 681–684. 29727194 10.1164/rccm.201801-0022LE PMC6118018

[B54] Goncharov D. A. Kudryashova T. V. Ziai H. Ihida-Stansbury K. DeLisser H. Krymskaya V. P. (2014). Mammalian target of rapamycin complex 2 (mTORC2) coordinates pulmonary artery smooth muscle cell metabolism, proliferation, and survival in pulmonary arterial hypertension. *Circulation* 129 864–874. 10.1161/circulationaha.113.004581 24270265 PMC3968690

[B55] Gowans G. J. Hawley S. A. Ross F. A. Hardie D. G. (2013). AMP is a true physiological regulator of AMP-activated protein kinase by both allosteric activation and enhancing net phosphorylation. *Cell Metab.* 18 556–566. 10.1016/j.cmet.2013.08.019 24093679 PMC3791399

[B56] Grimm D. J. Dawson C. A. Hakim T. S. Linehan J. H. (1978). Pulmonary vasomotion and the distribution of vascular resistance in a dog lung lobe. *J. Appl. Physiol.* 45 545–550. 10.1152/jappl.1978.45.4.545 711571

[B57] Gui D. Cui Z. Zhang L. Yu C. Yao D. Xu M. (2017). Salidroside attenuates hypoxia-induced pulmonary arterial smooth muscle cell proliferation and apoptosis resistance by upregulating autophagy through the AMPK-mTOR-ULK1 pathway. *BMC Pulmon. Med.* 17:191. 10.1186/s12890-017-0477-4 PMC5726034 29233105

[B58] Gwinn D. M. Shackelford D. B. Egan D. F. Mihaylova M. M. Mery A. Vasquez D. S. (2008). AMPK phosphorylation of raptor mediates a metabolic checkpoint. *Mol. Cell* 30 214–226. 10.1016/j.molcel.2008.03.003 18439900 PMC2674027

[B59] Hansmann G. Wagner R. A. Schellong S. Perez V. A. d. J. Urashima T. Wang L. (2007). Pulmonary arterial hypertension is linked to insulin resistance and reversed by peroxisome proliferator-activated receptor-gamma activation. *Circulation* 115 1275–1284. 10.1161/circulationaha.106.663120 17339547

[B60] Hardie D. G. (2008). AMPK: a key regulator of energy balance in the single cell and the whole organism. *Int. J. Obes.* 32 S7–S12. 10.1038/ijo.2008.116 18719601

[B61] Hardie D. G. (2013). AMPK: a target for drugs and natural products with effects on both diabetes and cancer. *Diabetes* 62 2164–2172. 10.2337/db13-0368 23801715 PMC3712072

[B62] Hardie D. G. Carling D. Gamblin S. J. (2011). AMP-activated protein kinase: also regulated by ADP? *Trends Biochem. Sci.* 36 470–477. 10.1016/j.tibs.2011.06.004 21782450

[B63] Hawley S. A. Boudeau J. Reid J. L. Mustard K. J. Udd L. Mäkelä T. P. (2003). Complexes between the LKB1 tumor suppressor, STRAD alpha/beta and MO25 alpha/beta are upstream kinases in the AMP-activated protein kinase cascade. *J. Biol.* 2:28. 10.1186/1475-4924-2-28 PMC333410 14511394

[B64] Hawley S. A. Pan D. A. Mustard K. J. Ross L. Bain J. Edelman A. M. (2005). Calmodulin-dependent protein kinase kinase-β is an alternative upstream kinase for AMP-activated protein kinase. *Cell Metab.* 2 9–19. 10.1016/j.cmet.2005.05.009 16054095

[B65] Heath D. Edwards J. E. (1958). The pathology of hypertensive pulmonary vascular disease. *Circulation* 18 533–547. 13573570 10.1161/01.cir.18.4.533

[B66] Herzig S. Shaw R. J. (2018). AMPK: guardian of metabolism and mitochondrial homeostasis. *Nat. Rev. Mol. Cell Biol.* 19 121–135. 10.1038/nrm.2017.95 28974774 PMC5780224

[B67] Hinchy E. C. Gruszczyk A. V. Willows R. Navaratnam N. Hall A. R. Bates G. (2018). Mitochondria-derived ROS activate AMP-activated protein kinase (AMPK) indirectly. *J. Biol. Chem.* 293 17208–17217. 10.1074/jbc.ra118.002579 30232152 PMC6222118

[B68] Hoeper M. M. Lam C. S. P. Vachiery J.-L. Bauersachs J. Gerges C. Lang I. M. (2016). Pulmonary hypertension in heart failure with preserved ejection fraction: a plea for proper phenotyping and further research. *Eur. Heart J.* 38 2869–2873. 10.1093/eurheartj/ehw597 28011705

[B69] Hong S.-P. Momcilovic M. Carlson M. (2005). Function of mammalian LKB1 and Ca2+/calmodulin-dependent protein kinase kinase α as Snf1-activating kinases in yeast. *J. Biol. Chem.* 280 21804–21809. 10.1074/jbc.m501887200 15831494

[B70] Huang X. Fan R. Lu Y. Yu C. Xu X. Zhang X. (2014). Regulatory effect of AMP-activated protein kinase on pulmonary hypertension induced by chronic hypoxia in rats: in vivo and in vitro studies. *Mol. Biol. Rep.* 41 4031–4041. 10.1007/s11033-014-3272-9 24566685

[B71] Hudson E. R. Pan D. A. James J. Lucocq J. M. Hawley S. A. Green K. A. (2003). A novel domain in AMP-activated protein kinase causes glycogen storage bodies similar to those seen in hereditary cardiac arrhythmias. *Curr. Biol.* 13 861–866. 10.1016/s0960-9822(03)00249-5 12747836

[B72] Humbert M. Guignabert C. Bonnet S. Dorfmüller P. Klinger J. R. Nicolls M. R. (2019). Pathology and pathobiology of pulmonary hypertension: state of the art and research perspectives. *Eur. Respirat. J.* 53:1801887. 10.1183/13993003.01887-2018 30545970 PMC6351340

[B73] Hurley R. L. Anderson K. A. Franzone J. M. Kemp B. E. Means A. R. Witters L. A. (2005). The Ca2+/calmodulin-dependent protein kinase kinases are AMP-activated protein kinase kinases. *J. Biol. Chem.* 280 29060–29066. 10.1074/jbc.m503824200 15980064

[B74] Ibe J. C. F. Zhou Q. Chen T. Tang H. Yuan J. X. J. Raj J. U. (2013). Adenosine monophosphate-activated protein kinase is required for pulmonary artery smooth muscle cell survival and the development of hypoxic pulmonary hypertension. *Am. J. Respirat. Cell Mol. Biol.* 49 609–618. 10.1165/rcmb.2012-0446oc 23668615 PMC3824043

[B75] Ido Y. Carling D. Ruderman N. (2002). Hyperglycemia-induced apoptosis in human umbilical vein endothelial cells: inhibition by the AMP-activated protein kinase activation. *Diabetes* 51 159–167. 10.2337/diabetes.51.1.159 11756336

[B76] Inoki K. Zhu T. Guan K.-L. (2003). TSC2 mediates cellular energy response to control cell growth and survival. *Cell* 115 577–590. 10.1016/s0092-8674(03)00929-2 14651849

[B77] Jasmin J. F. Lucas M. Cernacek P. Dupuis J. (2001). Effectiveness of a nonselective ET(A/B) and a selective ET(A) antagonist in rats with monocrotaline-induced pulmonary hypertension. *Circulation* 103 314–318. 10.1161/01.cir.103.2.314 11208695

[B78] Kahn B. B. Alquier T. Carling D. Hardie D. G. (2005). AMP-activated protein kinase: Ancient energy gauge provides clues to modern understanding of metabolism. *Cell Metab.* 1 15–25. 10.1016/j.cmet.2004.12.003 16054041

[B79] Kay J. M. Harris P. Heath D. (1967). Pulmonary hypertension produced in rats by ingestion of *Crotalaria spectabilis* seeds. *Thorax* 22 176–179. 10.1136/thx.22.2.176 6033385 PMC471603

[B80] Ke R. Liu L. Zhu Y. Li S. Xie X. Li F. (2016). Knockdown of AMPKα2 promotes pulmonary arterial smooth muscle cells proliferation via mTOR/Skp2/p27(Kip1) signaling pathway. *Int. J. Mol. Sci.* 17:844. 10.3390/ijms17060844 27258250 PMC4926378

[B81] Kelly M. Keller C. Avilucea P. R. Keller P. Luo Z. Xiang X. (2004). AMPK activity is diminished in tissues of IL-6 knockout mice: the effect of exercise. *Biochem. Biophys. Res. Commun.* 320 449–454. 10.1016/j.bbrc.2004.05.188 15219849

[B82] Kemp B. E. (2004). Bateman domains and adenosine derivatives form a binding contract. *J. Clin. Investigat.* 113 182–184. 10.1172/jci200420846 PMC311445 14722609

[B83] Kim A. S. Miller E. J. Wright T. M. Li J. Qi D. Atsina K. (2011). A small molecule AMPK activator protects the heart against ischemia–reperfusion injury. *J. Mol. Cell. Cardiol.* 51 24–32. 10.1016/j.yjmcc.2011.03.003 21402077 PMC4005884

[B84] Kim E. K. Lee J. H. Oh Y. M. Lee Y. S. Lee S. D. (2010). Rosiglitazone attenuates hypoxia-induced pulmonary arterial hypertension in rats. *Respirology (Carlton Vic.)* 15 659–668. 10.1111/j.1440-1843.2010.01756.x 20546541

[B85] Kim J. (2014). Apelin-APJ signaling: a potential therapeutic target for pulmonary arterial hypertension. *Mol. Cell.* 37 196–201. 10.14348/molcells.2014.2308 24608803 PMC3969039

[B86] Kim J. Yang G. Kim Y. Kim J. Ha J. (2016). AMPK activators: mechanisms of action and physiological activities. *Exp. Mol. Med.* 48:e224. 10.1038/emm.2016.16 27034026 PMC4855276

[B87] Klinger J. R. Abman S. H. Gladwin M. T. (2013). Nitric oxide deficiency and endothelial dysfunction in pulmonary arterial hypertension. *Am. J. Respirat. Crit. Care Med.* 188 639–646. 10.1164/rccm.201304-0686pp 23822809

[B88] Kola B. (2008). Role of AMP-activated protein kinase in the control of appetite. *J. Neuroendocrinol.* 20 942–951. 10.1111/j.1365-2826.2008.01745.x 18445126 PMC2658714

[B89] Kola B. Hubina E. Tucci S. A. Kirkham T. C. Garcia E. A. Mitchell S. E. (2005). Cannabinoids and ghrelin have both central and peripheral metabolic and cardiac effects via AMP-activated protein kinase. *J. Biol. Chem.* 280 25196–25201. 10.1074/jbc.c500175200 15899896

[B90] Koo S.-H. Flechner L. Qi L. Zhang X. Screaton R. A. Jeffries S. (2005). The CREB coactivator TORC2 is a key regulator of fasting glucose metabolism. *Nature* 437 1109–1114. 10.1038/nature03967 16148943

[B91] Krymskaya V. P. Snow J. Cesarone G. Khavin I. Goncharov D. A. Lim P. N. (2011). mTOR is required for pulmonary arterial vascular smooth muscle cell proliferation under chronic hypoxia. *FASEB J.* 25 1922–1933. 10.1096/fj.10-175018 21368105 PMC3101038

[B92] Lai Y. C. Tabima D. M. Dube J. J. Hughan K. S. Vanderpool R. R. Goncharov D. A. (2016). ESIRT3-AMP-activated protein kinase activation by nitrite and metformin improves hyperglycemia and normalizes pulmonary hypertension associated with heart failure with preserved ejection fraction. *Circulation* 133 717–731. 10.1161/circulationaha.115.018935 26813102 PMC4766041

[B93] Lamia K. A. Sachdeva U. M. DiTacchio L. Williams E. C. Alvarez J. G. Egan D. F. (2009). AMPK regulates the circadian clock by cryptochrome phosphorylation and degradation. *Science* 326 437–440. 10.1126/science.1172156 19833968 PMC2819106

[B94] Legchenko E. Chouvarine P. Borchert P. Fernandez-Gonzalez A. Snay E. Meier M. (2018). PPARγ agonist pioglitazone reverses pulmonary hypertension and prevents right heart failure via fatty acid oxidation. *Sci. Transl. Med.* 10:0303. 10.1126/scitranslmed.aao0303 29695452

[B95] Li M. Riddle S. R. Frid M. G. El Kasmi K. C. McKinsey T. A. Sokol R. J. (2011). Emergence of fibroblasts with a proinflammatory epigenetically altered phenotype in severe hypoxic pulmonary hypertension. *J. Immunol.* 187 2711–2722. 10.4049/jimmunol.1100479 21813768 PMC3159707

[B96] Li S. Han D. Zhang Y. Xie X. Ke R. Zhu Y. (2016). Activation of AMPK prevents monocrotaline-induced extracellular matrix remodeling of pulmonary artery. *Med. Sci. Monit. Basic Res.* 22 27–33. 10.12659/msmbr.897505 26978596 PMC4795089

[B97] Li Y. Xu S. Mihaylova M. M. Zheng B. Hou X. Jiang B. (2011). AMPK Phosphorylates and Inhibits SREBP activity to attenuate hepatic steatosis and atherosclerosis in diet-induced insulin-resistant mice. *Cell Metab.* 13 376–388. 10.1016/j.cmet.2011.03.009 21459323 PMC3086578

[B98] Liao S. Li D. Hui Z. McLachlan C. S. Zhang Y. (2018). Metformin added to bosentan therapy in patients with pulmonary arterial hypertension associated with congenital heart defects: a pilot study. *ERJ Open Res.* 4 00060–2018. 10.1183/23120541.00060-2018 30151369 PMC6104295

[B99] Liao S. Li D. Hui Z. McLachlan C. S. Zhang Y. (2019). Chronic dosing with metformin plus bosentan decreases in vitro pulmonary artery contraction from isolated arteries in adults with pulmonary hypertension. *J. Cardiovasc. Thorac. Res.* 11 189–195. 10.15171/jcvtr.2019.32 31579458 PMC6759611

[B100] Liu Y. Xu Y. Zhu J. Li H. Zhang J. Yang G. (2019). Metformin prevents progression of experimental pulmonary hypertension via inhibition of autophagy and activation of adenosine monophosphate-activated protein kinase. *J. Vascul. Res.* 56 117–128. 10.1159/000498894 31085922

[B101] Lizcano J. M. Göransson O. Toth R. Deak M. Morrice N. A. Boudeau J. (2004). LKB1 is a master kinase that activates 13 kinases of the AMPK subfamily, including MARK/PAR-1. *EMBO J.* 23 833–843. 10.1038/sj.emboj.7600110 14976552 PMC381014

[B102] Lv Y. Tang L.-L. Wei J.-K. Xu X.-F. Gu W. Fu L.-C. (2013). Decreased Kv1.5 expression in intrauterine growth retardation rats with exaggerated pulmonary hypertension. *Am. J. Physiol. Lung Cell. Mol. Physiol.* 305:L856. 10.1152/ajplung.00179.2013 24077947

[B103] Lyle M. A. Davis J. P. Brozovich F. V. (2017). Regulation of pulmonary vascular smooth muscle contractility in pulmonary arterial hypertension: implications for therapy. *Front. Physiol.* 8:614–614. 28878690 10.3389/fphys.2017.00614 PMC5572347

[B104] Ma H. Wang J. Thomas D. P. Tong C. Leng L. Wang W. (2010). Impaired macrophage migration inhibitory factor-AMP-activated protein kinase activation and ischemic recovery in the senescent heart. *Circulation* 122 282–292. 10.1161/circulationaha.110.953208 20606117 PMC2907453

[B105] Madden J. A. Dawson C. A. Harder D. R. (1985). Hypoxia-induced activation in small isolated pulmonary arteries from the cat. *J. Appl. Physiol.* 59 113–118. 10.1152/jappl.1985.59.1.113 4030552

[B106] Madden J. A. Vadula M. S. Kurup V. P. (1992). Effects of hypoxia and other vasoactive agents on pulmonary and cerebral artery smooth muscle cells. *Am. J. Physiol. Lung Cell. Mol. Physiol.* 263 L384–L393. 10.1152/ajplung.1992.263.3.L384 1415563

[B107] Maron B. A. Galiè N. (2016). Diagnosis, treatment, and clinical management of pulmonary arterial hypertension in the contemporary era: a review. *JAMA Cardiol.* 1 1056–1065. 10.1001/jamacardio.2016.4471 27851839 PMC5177491

[B108] Maron B. A. Leopold J. A. (2015). Emerging concepts in the molecular basis of pulmonary arterial hypertension: part II: neurohormonal signaling contributes to the pulmonary vascular and right ventricular pathophenotype of pulmonary arterial hypertension. *Circulation* 131 2079–2091. 10.1161/circulationaha.114.006980 26056345 PMC4465126

[B109] Marsin A. S. Bertrand L. Rider M. H. Deprez J. Beauloye C. Vincent M. F. (2000). Phosphorylation and activation of heart PFK-2 by AMPK has a role in the stimulation of glycolysis during ischaemia. *Curr. Biol.* 10 1247–1255. 10.1016/s0960-9822(00)00742-9 11069105

[B110] McMurtry I. F. Davidson A. B. Reeves J. T. Grover R. F. (1976). Inhibition of hypoxic pulmonary vasoconstriction by calcium antagonists in isolated rat lungs. *Circulat. Res.* 38 99–104. 10.1161/01.res.38.2.99 1245025

[B111] Mihaylova M. M. Shaw R. J. (2011). The AMPK signalling pathway coordinates cell growth, autophagy and metabolism. *Nat. Cell Biol.* 13 1016–1023. 10.1038/ncb2329 21892142 PMC3249400

[B112] Miller E. J. Li J. Leng L. McDonald C. Atsumi T. Bucala R. (2008). Macrophage migration inhibitory factor stimulates AMP-activated protein kinase in the ischaemic heart. *Nature* 451 578–582. 10.1038/nature06504 18235500

[B113] Momcilovic M. Hong S.-P. Carlson M. (2006). Mammalian TAK1 activates Snf1 protein kinase in yeast and phosphorylates AMP-activated protein kinase in vitro. *J. Biol. Chem.* 281 25336–25343. 10.1074/jbc.m604399200 16835226

[B114] Moral-Sanz J. Lewis S. A. MacMillan S. Ross F. A. Thomson A. Viollet B. (2018). The LKB1–AMPK-α1 signaling pathway triggers hypoxic pulmonary vasoconstriction downstream of mitochondria. *Sci. Signal.* 11:0296. 10.1126/scisignal.aau0296 30279167

[B115] Moral-Sanz J. Mahmoud A. D. Ross F. A. Eldstrom J. Fedida D. Hardie D. G. (2016). AMP-activated protein kinase inhibits Kv 1.5 channel currents of pulmonary arterial myocytes in response to hypoxia and inhibition of mitochondrial oxidative phosphorylation. *J. Physiol.* 594 4901–4915. 10.1113/jp272032 27062501 PMC5009768

[B116] Morrison A. Yan X. Tong C. Li J. (2011). Acute rosiglitazone treatment is cardioprotective against ischemia-reperfusion injury by modulating AMPK, Akt, and JNK signaling in nondiabetic mice. *Am. J. Physiol. Heart Circulat. Physiol.* 301 H895–H902. 10.1152/ajpheart.00137.2011 21666107

[B117] Moudgil R. Michelakis E. D. Archer S. L. (2005). Hypoxic pulmonary vasoconstriction. *J. Appl. Physiol.* 98 390–403. 15591309 10.1152/japplphysiol.00733.2004

[B118] Munday M. R. Campbell D. G. Carling D. Hardie D. G. (1988). Identification by amino acid sequencing of three major regulatory phosphorylation sites on rat acetyl-CoA carboxylase. *Eur. J. Biochem.* 175 331–338. 10.1111/j.1432-1033.1988.tb14201.x 2900138

[B119] Mungai P. T. Waypa G. B. Jairaman A. Prakriya M. Dokic D. Ball M. K. (2011). Hypoxia triggers AMPK Activation through reactive oxygen species-mediated activation of calcium release-activated calcium channels. *Mol. Cell. Biol.* 31 3531–3545. 10.1128/mcb.05124-11 21670147 PMC3165558

[B120] Murray T. R. Chen L. Marshall B. E. Macarak E. J. (1990a). Hypoxic contraction of cultured pulmonary vascular smooth muscle cells. *Am. J. Respirat. Cell Mol. Biol.* 3 457–465. 10.1165/ajrcmb/3.5.457 2223100

[B121] Murray T. R. Marshall B. E. Macarak E. J. (1990b). Contraction of vascular smooth muscle in cell culture. *J. Cell. Physiol.* 143 26–38. 10.1007/978-3-642-66427-4_6 2318908

[B122] Myers R. W. Guan H.-P. Ehrhart J. Petrov A. Prahalada S. Tozzo E. (2017). pan-AMPK activator MK-8722 improves glucose homeostasis but induces cardiac hypertrophy. *Science* 357 507–511. 10.1126/science.aah5582 28705990

[B123] Nagata D. Kiyosue A. Takahashi M. Satonaka H. Tanaka K. Sata M. (2009). A new constitutively active mutant of AMP-activated protein kinase inhibits anoxia-induced apoptosis of vascular endothelial cell. *Hypertens. Res.* 32 133–139. 10.1038/hr.2008.25 19262472

[B124] Nicolls M. R. Voelkel N. F. (2017). The roles of immunity in the prevention and evolution of pulmonary arterial hypertension. *Am. J. Respirat. Crit. Care Med.* 195 1292–1299. 10.1164/rccm.201608-1630pp 27786553 PMC5443903

[B125] Oakhill J. S. Chen Z.-P. Scott J. W. Steel R. Castelli L. A. Ling N. (2010). β-Subunit myristoylation is the gatekeeper for initiating metabolic stress sensing by AMP-activated protein kinase (AMPK). *Proc. Natl. Acad. Sci. U.S.A.* 107 19237–19241. 10.1073/pnas.1009705107 20974912 PMC2984171

[B126] Oakhill J. S. Scott J. W. Kemp B. E. (2012). AMPK functions as an adenylate charge-regulated protein kinase. *Trends Endocrinol. Metabol.* 23 125–132. 10.1016/j.tem.2011.12.006 22284532

[B127] Omura J. Satoh K. Kikuchi N. Satoh T. Kurosawa R. Nogi M. (2016). Protective roles of endothelial AMP-activated protein kinase against hypoxia-induced pulmonary hypertension in mice. *Circulat. Res.* 119 197–209. 10.1161/circresaha.115.308178 27217398

[B128] Park J. W. Lee J. H. Park Y. H. Park S. J. Cheon J. H. Kim W. H. (2017). Sex-dependent difference in the effect of metformin on colorectal cancer-specific mortality of diabetic colorectal cancer patients. *World J. Gastroenterol.* 23 5196–5205. 10.3748/wjg.v23.i28.5196 28811714 PMC5537186

[B129] Penaloza D. Arias-Stella J. (2007). The heart and pulmonary circulation at high altitudes. *Circulation* 115 1132–1146. 10.1161/circulationaha.106.624544 17339571

[B130] Plecitá-Hlavatá L. Tauber J. Li M. Zhang H. Flockton A. R. Pullamsetti S. S. (2016). Constitutive reprogramming of fibroblast mitochondrial metabolism in pulmonary hypertension. *Am. J. Respirat. Cell Mol. Biol.* 55 47–57. 10.1165/rcmb.2015-0142oc 26699943 PMC4942204

[B131] Quan H. Zhang H. Wei W. Fang T. (2016). Gender-related different effects of a combined therapy of Exenatide and Metformin on overweight or obesity patients with type 2 diabetes mellitus. *J. Diab. Complicat.* 30 686–692. 10.1016/j.jdiacomp.2016.01.013 26873871

[B132] Rabinovitch M. Guignabert C. Humbert M. Nicolls M. R. (2014). Inflammation and immunity in the pathogenesis of pulmonary arterial hypertension. *Circulat. Res.* 115 165–175. 10.1161/circresaha.113.301141 24951765 PMC4097142

[B133] Rana U. Callan E. Entringer B. Michalkiewicz T. Joshi A. Parchur A. K. (2020). AMP-kinase dysfunction alters notch ligands to impair angiogenesis in neonatal pulmonary hypertension. *Am. J. Respirat. Cell Mol. Biol.* 62 719–731. 10.1165/rcmb.2019-0275oc 32048878 PMC7258820

[B134] Ranchoux B. Harvey L. D. Ayon R. J. Babicheva A. Bonnet S. Chan S. Y. (2018). Endothelial dysfunction in pulmonary arterial hypertension: an evolving landscape (2017 Grover Conference Series). *Pulmon. Circulat.* 8:2045893217752912. 10.1177/2045893217752912 PMC5798691 29283043

[B135] Robertson T. P. Mustard K. J. Lewis T. H. Clark J. H. Wyatt C. N. Blanco E. A. (2008). AMP-activated protein kinase and hypoxic pulmonary vasoconstriction. *Eur. J. Pharmacol.* 595 39–43. 10.1016/j.ejphar.2008.07.035 18703047 PMC3119428

[B136] Rojas J. Arraiz N. Aguirre M. Velasco M. Bermúdez V. (2011). AMPK as target for intervention in childhood and adolescent obesity. *Int. J. Obesit.* 2011 252817–252817. 10.1155/2011/252817 PMC3034972 21318055

[B137] Ross F. A. Jensen T. E. Hardie D. G. (2016a). Differential regulation by AMP and ADP of AMPK complexes containing different γ subunit isoforms. *Biochem. J.* 473 189–199. 10.1042/bj20150910 26542978 PMC4700476

[B138] Ross F. A. MacKintosh C. Hardie D. G. (2016b). AMP-activated protein kinase: a cellular energy sensor that comes in 12 flavours. *FEBS J.* 283 2987–3001. 10.1111/febs.13698 26934201 PMC4995730

[B139] Ruderman N. B. Park H. Kaushik V. K. Dean D. Constant S. Prentki M. (2003). AMPK as a metabolic switch in rat muscle, liver and adipose tissue after exercise. *Acta Physiol. Scand.* 178 435–442. 10.1046/j.1365-201x.2003.01164.x 12864749

[B140] Russell R. R. I. I. I. Li J. Coven D. L. Pypaert M. Zechner C. Palmeri M. (2004). AMP-activated protein kinase mediates ischemic glucose uptake and prevents postischemic cardiac dysfunction, apoptosis, and injury. *J. Clin. Investigat.* 114 495–503. 10.1172/jci19297 15314686 PMC503766

[B141] Rutter G. A. da Silva Xavier G. Leclerc I. (2003). Roles of 5′-AMP-activated protein kinase (AMPK) in mammalian glucose homoeostasis. *Biochem. J.* 375 1–16. 10.1042/bj20030048 12839490 PMC1223661

[B142] Ryan J. J. Marsboom G. Archer S. L. (2013). Rodent models of group 1 pulmonary hypertension. *Handbook Exp. Pharmacol.* 218 105–149. 10.1007/978-3-662-45805-1_5 24092338

[B143] Sakamoto K. McCarthy A. Smith D. Green K. A. Hardie D. G. Ashworth A. (2005). Deficiency of LKB1 in skeletal muscle prevents AMPK activation and glucose uptake during contraction. *EMBO J.* 24 1810–1820. 10.1038/sj.emboj.7600667 15889149 PMC1142598

[B144] Sakamoto K. Zarrinpashneh E. Budas G. R. Pouleur A. C. Dutta A. Prescott A. R. (2006). Deficiency of LKB1 in heart prevents ischemia-mediated activation of AMPKalpha2 but not AMPKalpha1. *Am. J. Physiol. Endocrinol. Metabol.* 290 E780–E788. 10.1152/ajpendo.00443.2005 PMC2128705 16332922

[B145] Sakao S. Tatsumi K. (2011). The effects of antiangiogenic compound SU5416 in a rat model of pulmonary arterial hypertension. *Respirat. Int. Rev. Thorac. Dis.* 81 253–261. 10.1159/000322011 21116108

[B146] Sallé-Lefort S. Miard S. Nolin M.-A. Boivin L. Paré M. -È Debigaré R. (2016). Hypoxia upregulates Malat1 expression through a CaMKK/AMPK/HIF-1α axis. *Int. J. Oncol.* 49 1731–1736. 10.3892/ijo.2016.3630 27499160

[B147] Satoh K. Fukumoto Y. Nakano M. Sugimura K. Nawata J. Demachi J. (2009). Statin ameliorates hypoxia-induced pulmonary hypertension associated with down-regulated stromal cell-derived factor-1. *Cardiovasc. Res.* 81 226–234. 10.1093/cvr/cvn244 18779230

[B148] Schoental R. Head M. A. (1955). Pathological changes in rats as a result of treatment with monocrotaline. *Br. J. Cancer* 9 229–237. 10.1038/bjc.1955.19 14378507 PMC2073973

[B149] Scott J. W. Norman D. G. Hawley S. A. Kontogiannis L. Hardie D. G. (2002). Protein kinase substrate recognition studied using the recombinant catalytic domain of AMP-activated protein kinase and a model substrate. *J. Mol. Biol.* 317 309–323. 10.1006/jmbi.2001.5316 11902845

[B150] Shackelford D. B. Shaw R. J. (2009). The LKB1-AMPK pathway: metabolism and growth control in tumour suppression. *Nat. Rev. Cancer* 9 563–575. 10.1038/nrc2676 19629071 PMC2756045

[B151] Shaw R. J. Kosmatka M. Bardeesy N. Hurley R. L. Witters L. A. DePinho R. A. (2004). The tumor suppressor LKB1 kinase directly activates AMP-activated kinase and regulates apoptosis in response to energy stress. *Proc. Natl. Acad. Sci. U.S.A.* 101 3329–3335. 10.1073/pnas.0308061100 14985505 PMC373461

[B152] Shaw R. J. Lamia K. A. Vasquez D. Koo S. H. Bardeesy N. Depinho R. A. (2005). The kinase LKB1 mediates glucose homeostasis in liver and therapeutic effects of metformin. *Science* 310 1642–1646. 10.1126/science.1120781 16308421 PMC3074427

[B153] Shimoda L. A. Laurie S. S. (2013). Vascular remodeling in pulmonary hypertension. *J. Mol. Med.* 91 297–309. 23334338 10.1007/s00109-013-0998-0 PMC3584237

[B154] Shirwany N. A. Zou M. H. (2010). AMPK in cardiovascular health and disease. *Acta Pharmacol. Sin.* 31 1075–1084. 10.1038/aps.2010.139 20711221 PMC3078651

[B155] Simonneau G. Montani D. Celermajer D. S. Denton C. P. Gatzoulis M. A. Krowka M. (2019). Haemodynamic definitions and updated clinical classification of pulmonary hypertension. *Eur. Respirat. J.* 53:1801913. 10.1183/13993003.01913-2018 30545968 PMC6351336

[B156] Sommer N. Dietrich A. Schermuly R. T. Ghofrani H. A. Gudermann T. Schulz R. (2008). Regulation of hypoxic pulmonary vasoconstriction: basic mechanisms. *Eur. Respirat. J.* 32 1639–1651. 19043010 10.1183/09031936.00013908

[B157] Song Y. Wu Y. Su X. Zhu Y. Liu L. Pan Y. (2016). Activation of AMPK inhibits PDGF-induced pulmonary arterial smooth muscle cells proliferation and its potential mechanisms. *Pharmacol. Res.* 107 117–124. 10.1016/j.phrs.2016.03.010 26993101

[B158] Stapleton D. Mitchelhill K. I. Gao G. Widmer J. Michell B. J. Teh T. (1996). Mammalian AMP-activated protein kinase subfamily. *J. Biol. Chem.* 271 611–614. 8557660 10.1074/jbc.271.2.611

[B159] Stenmark K. R. McMurtry I. F. (2005). Vascular remodeling versus vasoconstriction in chronic hypoxic pulmonary hypertension. *Circul. Res.* 97 95–98. 10.1161/01.res.00000175934.68087.29 16037575

[B160] Stenmark K. R. Fagan K. A. Frid M. G. (2006). Hypoxia-induced pulmonary vascular remodeling. *Circulat. Res.* 99 675–691. 17008597 10.1161/01.RES.0000243584.45145.3f

[B161] Sun Z. Liu Y. Yu F. Xu Y. Yanli L. Liu N. (2019). Long non-coding RNA and mRNA profile analysis of metformin to reverse the pulmonary hypertension vascular remodeling induced by monocrotaline. *Biomed. Pharmacother.* 115:108933. 10.1016/j.biopha.2019.108933 31060005

[B162] Sylvester J. T. Shimoda L. A. Aaronson P. I. Ward J. P. T. (2012). Hypoxic pulmonary vasoconstriction. *Physiol. Rev.* 92 367–520. 22298659 10.1152/physrev.00041.2010 PMC9469196

[B163] Sztuka K. Jasiñska-Stroschein M. (2017). Animal models of pulmonary arterial hypertension: a systematic review and meta-analysis of data from 6126 animals. *Pharmacol. Res.* 125 201–214. 10.1016/j.phrs.2017.08.003 28867639

[B164] Taraseviciene-Stewart L. Kasahara Y. Alger L. Hirth P. Mc Mahon G. Waltenberger J. (2001). Inhibition of the VEGF receptor 2 combined with chronic hypoxia causes cell death-dependent pulmonary endothelial cell proliferation and severe pulmonary hypertension. *FASEB J.* 15 427–438. 10.1096/fj.00-0343com 11156958

[B165] Tarry D. Powell M. (2017). Hypoxic pulmonary vasoconstriction. *BJA Educat.* 17 208–213.

[B166] Teng R.-J. Du J. Afolayan A. J. Eis A. Shi Y. Konduri G. G. (2013). AMP kinase activation improves angiogenesis in pulmonary artery endothelial cells with in utero pulmonary hypertension. *Am. J. Physiol. Lung Cell. Mol. Physiol.* 304 L29–L42. 23103561 10.1152/ajplung.00200.2012 PMC3543642

[B167] Thenappan T. Ormiston M. L. Ryan J. J. Archer S. L. (2018). Pulmonary arterial hypertension: pathogenesis and clinical management. *BMJ* 360:j5492. 10.1136/bmj.j5492 29540357 PMC6889979

[B168] Thenappan T. Shah S. J. Gomberg-Maitland M. Collander B. Vallakati A. Shroff P. (2011). Clinical characteristics of pulmonary hypertension in patients with heart failure and preserved ejection fraction. *Circulat. Heart Fail.* 4 257–265. 10.1161/CIRCHEARTFAILURE.110.958801 21411741

[B169] Thomson D. M. Porter B. B. Tall J. H. Kim H.-J. Barrow J. R. Winder W. W. (2007). Skeletal muscle and heart LKB1 deficiency causes decreased voluntary running and reduced muscle mitochondrial marker enzyme expression in mice. *Am. J. Physiol. Endocrinol. Metab.* 292 E196–E202. 16926377 10.1152/ajpendo.00366.2006

[B170] Towler M. C. Hardie D. G. (2007). AMP-activated protein kinase in metabolic control and insulin signaling. *Circulat. Res.* 100 328–341. 10.1161/01.res.0000256090.42690.05 17307971

[B171] Toyama E. Q. Herzig S. Courchet J. Lewis T. L. Losón O. C. Hellberg K. (2016). AMP-activated protein kinase mediates mitochondrial fission in response to energy stress. *Science* 351 275–281. 10.1126/science.aab4138 26816379 PMC4852862

[B172] Tuder R. M. (2017). Pulmonary vascular remodeling in pulmonary hypertension. *Cell Tissue Res.* 367 643–649. 28025704 10.1007/s00441-016-2539-y PMC5408737

[B173] Tuder R. M. Marecki J. C. Richter A. Fijalkowska I. Flores S. (2007). Pathology of pulmonary hypertension. *Clin. Chest Med.* 28 23–vii. 17338926 10.1016/j.ccm.2006.11.010 PMC1924722

[B174] Viollet B. Lantier L. Devin-Leclerc J. Hebrard S. Amouyal C. Mounier R. (2009). Targeting the AMPK pathway for the treatment of Type 2 diabetes. *Front. Biosci. (Landmark Ed.)* 14:3380–3400. 10.2741/3460 19273282 PMC2677695

[B175] von Euler U. S. von Liljestrand G. (1946). Observations on the pulmonary arterial blood pressure in the cat. *Acta Physiol. Scandinav.* 12 301–320. 10.1111/j.1748-1716.1946.tb00389.x

[B176] Wang L. Halliday G. Huot J. R. Satoh T. Baust J. J. Fisher A. (2020). Treatment with treprostinil and metformin normalizes hyperglycemia and improves cardiac function in pulmonary hypertension associated with heart failure with preserved ejection fraction. *Arterioscl. Thromb. Vascul. Biol.* 40 1543–1558. 10.1161/atvbaha.119.313883 32268788 PMC7255946

[B177] Wang Y. -g. Han X.-g. Yang Y. Qiao H. Dai K. -r. Fan Q. -m. (2016). Functional differences between AMPK α1 and α2 subunits in osteogenesis, osteoblast-associated induction of osteoclastogenesis, and adipogenesis. *Sci. Rep.* 6:32771. 10.1038/srep32771 PMC5013406 27600021

[B178] Wang Z. Wilson W. A. Fujino M. A. Roach P. J. (2001). Antagonistic controls of autophagy and glycogen accumulation by Snf1p, the yeast homolog of AMP-activated protein kinase, and the cyclin-dependent kinase Pho85p. *Mol. Cell. Biol.* 21 5742–5752. 10.1128/mcb.21.17.5742-5752.2001 11486014 PMC87294

[B179] Waypa G. B. Schumacker P. T. (2010). Hypoxia-induced changes in pulmonary and systemic vascular resistance: where is the O2 sensor. *Respirat. Physiol. Neurobiol.* 174 201–211. 10.1016/j.resp.2010.08.007 20713189 PMC2991475

[B180] Weir E. K. Archer S. L. (1995). The mechanism of acute hypoxic pulmonary vasoconstriction: the tale of two channels. *FASEB J.* 9 183–189. 10.1096/fasebj.9.2.7781921 7781921

[B181] Wilson D. W. Segall H. J. Pan L. C. Dunston S. K. (1989). Progressive inflammatory and structural changes in the pulmonary vasculature of monocrotaline-treated rats. *Microvasc. Res.* 38 57–80. 10.1016/0026-2862(89)90017-4 2503687

[B182] Woods A. Dickerson K. Heath R. Hong S. P. Momcilovic M. Johnstone S. R. (2005). Ca2+/calmodulin-dependent protein kinase kinase-beta acts upstream of AMP-activated protein kinase in mammalian cells. *Cell Metab.* 2 21–33. 10.1016/j.cmet.2005.06.005 16054096

[B183] Woods A. Johnstone S. R. Dickerson K. Leiper F. C. Fryer L. G. Neumann D. (2003). LKB1 is the upstream kinase in the AMP-activated protein kinase cascade. *Curr. Biol.* 13 2004–2008. 10.1016/j.cub.2003.10.031 14614828

[B184] Wu N. Zheng B. Shaywitz A. Dagon Y. Tower C. Bellinger G. (2013). AMPK-dependent degradation of TXNIP upon energy stress leads to enhanced glucose uptake via GLUT1. *Mol. Cell* 49 1167–1175. 10.1016/j.molcel.2013.01.035 23453806 PMC3615143

[B185] Wu S. Zou M. H. (2020). AMPK, mitochondrial function, and cardiovascular disease. *Int. J. Mol. Sci.* 21:4987. 10.3390/ijms21144987 32679729 PMC7404275

[B186] Wu Y. Liu L. Zhang Y. Wang G. Han D. Ke R. (2014). Activation of AMPK inhibits pulmonary arterial smooth muscle cells proliferation. *Exp. Lung Res.* 40 251–258. 10.3109/01902148.2014.913092 24809794

[B187] Wu Y. Song P. Zhang W. Liu J. Dai X. Liu Z. (2015). Activation of AMPKα2 in adipocytes is essential for nicotine-induced insulin resistance in vivo. *Nat. Med.* 21 373–382. 10.1038/nm.3826 25799226 PMC4390501

[B188] Xiao B. Heath R. Saiu P. Leiper F. C. Leone P. Jing C. (2007). Structural basis for AMP binding to mammalian AMP-activated protein kinase. *Nature* 449 496–500. 10.1038/nature06161 17851531

[B189] Xiao B. Sanders M. J. Underwood E. Heath R. Mayer F. V. Carmena D. (2011). Structure of mammalian AMPK and its regulation by ADP. *Nature* 472 230–233. 10.1038/nature09932 21399626 PMC3078618

[B190] Xue J. Nelin L. D. Chen B. (2017). Hypoxia induces arginase II expression and increases viable human pulmonary artery smooth muscle cell numbers via AMPKα1 signaling. *Am. J. Physiol. Lung Cell. Mol. Physiol.* 312 L568–L578. 28213467 10.1152/ajplung.00117.2016 PMC5407096

[B191] Yan H. Zhang D. X. Shi X. Zhang Q. Huang Y. S. (2012). Activation of the prolyl-hydroxylase oxygen-sensing signal cascade leads to AMPK activation in cardiomyocytes. *J. Cell. Mol. Med.* 16 2049–2059. 10.1111/j.1582-4934.2011.01500.x 22128786 PMC3822975

[B192] Yan Y. Zhou X. E. Xu H. E. Melcher K. (2018). Structure and physiological regulation of AMPK. *Int. J. Mol. Sci.* 19:3534. 10.3390/ijms19113534 30423971 PMC6274893

[B193] Yoshida T. Matsuura K. Goya S. Ma D. Shimada K. Kitpipatkun P. (2020). Metformin prevents the development of monocrotaline-induced pulmonary hypertension by decreasing serum levels of big endothelin-1. *Exp. Therap. Med.* 20:149. 10.3892/etm.2020.9278 PMC7571338 33093887

[B194] Yuan J. X.-J. Aldinger A. M. Juhaszova M. Wang J. Conte J. V. Gaine S. P. (1998). Dysfunctional voltage-gated K+ channels in pulmonary artery smooth muscle cells of patients with primary pulmonary hypertension. *Circulation* 98 1400–1406. 10.1161/01.cir.98.14.1400 9760294

[B195] Zarrinpashneh E. Carjaval K. Beauloye C. Ginion A. Mateo P. Pouleur A.-C. (2006). Role of the α2-isoform of AMP-activated protein kinase in the metabolic response of the heart to no-flow ischemia. *Am. J. Physiol. Heart Circulat. Physiol.* 291 H2875–H2883. 10.1152/ajpheart.01032.2005 16877552

[B196] Zhai C. Shi W. Feng W. Zhu Y. Wang J. Li S. (2018). Activation of AMPK prevents monocrotaline-induced pulmonary arterial hypertension by suppression of NF-κB-mediated autophagy activation. *Life Sci.* 208 87–95. 10.1016/j.lfs.2018.07.018 30009823

[B197] Zhang J. Dong J. Martin M. He M. Gongol B. Marin T. L. (2018). Shyy, AMP-activated protein kinase phosphorylation of angiotensin-converting enzyme 2 in endothelium mitigates pulmonary hypertension. *Am. J. Respirat. Cell Mol. Biol.* 198 509–520. 10.1164/rccm.201712-2570oc 29570986 PMC6118028

[B198] Zhang Y. Lee T. S. Kolb E. M. Sun K. Lu X. Sladek F. M. (2006). AMP-activated protein kinase is involved in endothelial NO synthase activation in response to shear stress. *Arterioscler. Thromb. Vasc. Biol.* 26 1281–1287. 10.1161/01.atv.0000221230.08596.98 16601232

[B199] Zhao F. Wang C. Zhu X. (2020). Isoform-specific roles of AMPK catalytic α subunits in Alzheimer’s disease. *J. Clin. Investigat.* 130 3403–3405. 10.1172/jci137908 32452835 PMC7324169

[B200] Zhao L. (2010). Chronic hypoxia-induced pulmonary hypertension in rat: the best animal model for studying pulmonary vasoconstriction and vascular medial hypertrophy. *Drug Discover. Today Dis. Models* 7 83–88.

[B201] Zmijewski J. W. Banerjee S. Bae H. Friggeri A. Lazarowski E. R. Abraham E. (2010). Exposure to hydrogen peroxide induces oxidation and activation of AMP-activated protein kinase. *J. Biol. Chem.* 285 33154–33164. 10.1074/jbc.m110.143685 20729205 PMC2963401

[B202] Zong H. Ren J. M. Young L. H. Pypaert M. Mu J. Birnbaum M. J. (2002). AMP kinase is required for mitochondrial biogenesis in skeletal muscle in response to chronic energy deprivation. *Proc. Natl. Acad. Sci. U.S.A.* 99 15983–15987. 10.1073/pnas.252625599 12444247 PMC138551

